# Traditional lore on the healing effects of therapeutic plants used by the local communities around Simien Mountains National Park, northwestern Ethiopia

**DOI:** 10.1186/s13002-024-00678-9

**Published:** 2024-04-17

**Authors:** Endalkachew Seraw, Yirgalem Melkamu, Getinet Masresha

**Affiliations:** Biology Department, University of Gondar, P.O. Box 196, Gondar, Ethiopia

**Keywords:** Conservation, Indigenous knowledge, Traditional practitioners, Simien Mountain National Park, Traditional medicine

## Abstract

**Background:**

Simien Mountain National Park is a world heritage site with spectacular landscapes and rich in floral diversity**.** Exploring the plethora of conventional wisdom regarding therapeutic flora for sustainable use and drug development is a timely endeavor**.** Thus, the present study was aimed at investigating therapeutic plant uses and conservation practices by the local communities dwelling in the vicinity of the Park.

**Methods:**

Eighty randomly selected general informants and 20 purposefully selected key informants were used to collect the traditional lore from 10 purposefully selected kebeles that border the Park. Data were collected using face-to-face interviews, guided field walks, group discussions and market surveys. Descriptive statistics were used to analyze the basic information collected from the informants. An independent sample t test was computed to compare the knowledge variations among different informant groups. Clustering and ranking techniques were employed to validate traditional wisdom of informants.

**Results:**

Significant differences in traditional wisdom (*P* < 0.05) were observed only between general and key informants. Hundred thirteen therapeutic plant species belonging to 56 families were recorded. Asteraceae was the most species-rich family (10%). The majority of therapeutic species were collected from the wild (77%). Herbs and roots were the most preferred habit (47%) and plant parts (37%) for remedy preparation, respectively. Pounding was the most common preparation method (50.1%). The most frequently practiced route of administration was the oral route (48.1%). The highest Informant Consensus Factor (ICF) value (84%) was recorded for respiratory and febrile illnesses. *Rumex nepalensis* was the most preferred for the treatment of wounds, and *Olea europaea* subsp *cuspidata* was the first-ranked multipurpose plant.

**Conclusion:**

The Park is rich in therapeutic species serving as a refuge for many endemic and endangered species associated with the local community rich medicinal traditional lore. Erosion of therapeutic plants, verbal transfer of the traditional wisdom and young generation negligence in acquiring traditional lore led to the deterioration of the long tradition of using therapeutic plants for health care. Endangered multipurpose therapeutic plants like *Echinops kebericho* should get conservation priority. Therapeutic plants with the highest ICF and fidelity level could be candidates for drug development.

## Introduction

Starting from the time immemorial, local communities in Africa exercise traditional medicine as the primary healthcare system over modern medicines due to its affordability, acceptability and easy access to traditional plants [1]. In East Africa, including Ethiopia, traditional medicine is the dominant and most popular system of health care, used by about 80% of the population [2]. Matching to this, Ethiopia harbors a great diversity of geology, land forms, soils and climate that makes it a floristically diverse nation [3] that favors medicinal plant growth and utilization. As a result, Ethiopians are largely relied on their natural environments for their basic needs and health care. Thus, use of medicinal plants for health care is the long tradition of the Ethiopian people.

However, the documented medicinal plants in the country are limited compared to the floral diversity and the existing multiethnic and cultural diversity of the people as well as the long history of using medicinal plant for health care [4]. On the other side, the indigenous knowledge is passed verbally from generation to generation that can be easily deleted from the mind. In addition, medicinal plants and the associated knowledge are being seriously depleted due to deforestation, environmental degradation and acculturation that have been taking place in the country for quite a long time [5, 6]. This could ultimately result in the weakening of primary healthcare services.

Simien Mountains National Park (SMNP) and the surrounding areas are magnificent and spectacular land escapes. In honor of its exceptional biophysical features and unique combination of biodiversity with many endemic and endangered species, the park was inscribed on the UNESCO World Heritage List in 1978 [7]. Taking its international significance into consideration, except for the traditional lore on therapeutic plant species, a great deal of information has been published about the park as it draws the attention of scholars from various fields [8–14].

However, the park, like other regions of the nation, is still challenged by several anthropologic factors, such as overgrazing, agricultural encroachment and selective removal of plant species (SMNP Office report, 2020). In addition, SMNP is much more sensitive and vulnerable to the impacts of climate change since the park is included in the afromontane and afroalpine ecosystems, which are exceptionally vulnerable to climate change. Rising temperatures, changing rainfall patterns and an increased frequency of extreme weather events are affecting the park's ecosystems, including the distribution and survival of plant and animal species. Though the communities around the park have a long tradition of using therapeutic plants for their health care, the loss of indigenous flora and therapeutic plants with the associated indigenous lore is, also, one of the major challenges facing the park. Before the accelerated ecological and cultural transformation totally distorted the physical entities and the associated knowledge base, scientific documentation of traditional lore with therapeutic plants was a timely endeavor. It was expected that SMNP, with its vast microhabitats caused by its extraordinary topographic variability, would call for new exploration and recording of therapeutic plants, which could be commendable for this work. Therefore, the objectives of the present work were: (a) to document the long tradition of using therapeutic plants for health care; (b) to assess major threats to therapeutic plants; (c) to document the traditional practices of conserving therapeutic plants; and (d) to identify novel practices regarding the use of therapeutic plants among the residents living in the vicinity of the park.

## Materials and methods

### Descriptions of the study area

**Fig. 1:**
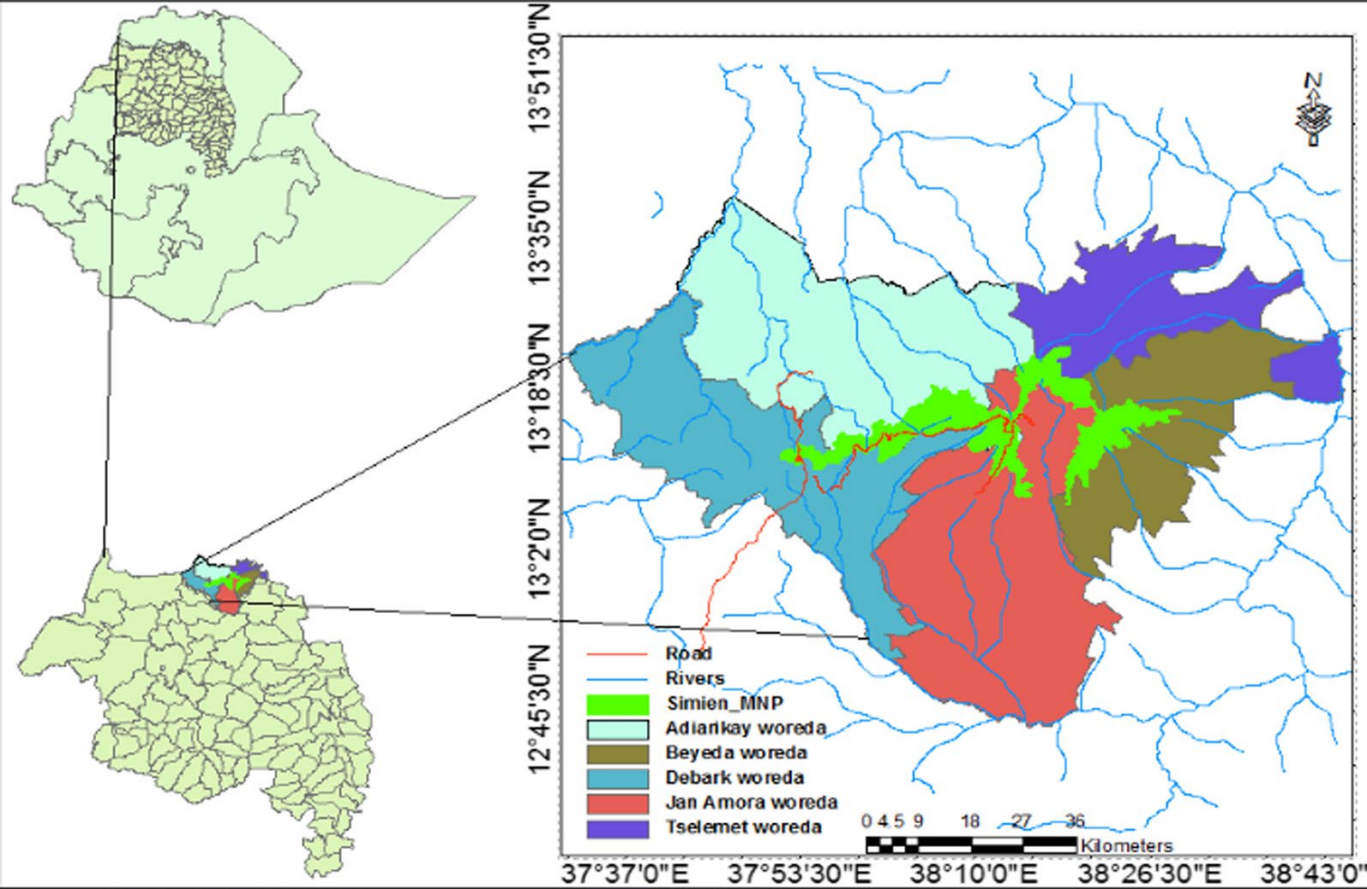
Location map of SMNP with the adjoining districts

The study was conducted in the vicinities of SMNP. SMNP is situated 857 km northwest of Addis Ababa, the capital of the nation. It was established in 1966 (SMNP Office report, 2020) and currently covers an area of 412 km^2^ [15]. It lies within five districts (Debark, Adiarkay, Janamora, Beyeda and Tellemit) and borders 42 kebeles stretching from 13° 06′ 44.09'' N to 13° 23′ 07.85'' N latitude and from 37° 51′26.36''E to 38° 29′ 27.59'' E longitude (Fig. 1).

**Fig. 2:**
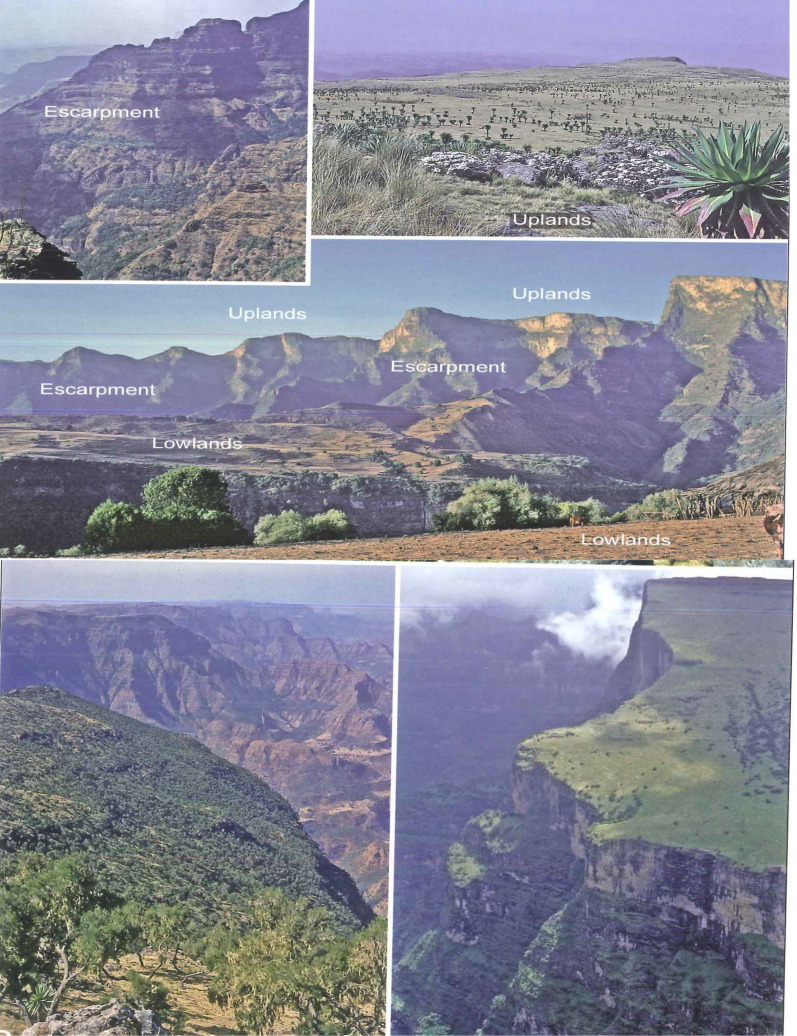
Different land forms of Simien Mountains

The Park is the highest regions of the Ethiopian Plateau, extending from 2000 to over 4,550 m a.s.l., i.e., Ras Dejen Mount, Ethiopia's highest peak point [16]. The park is an area of great diversity and scenic beauty and referred to as the “roof of Africa.” The unique geological formation of the mountains with the extensive erosion of the basaltic layers over a long period of time led to the formation of different land forms that make up the spectacular landscape of SMNP (Fig. 2). The presence of unique landscapes and biodiversity made SMNP a World Heritage Site in 1978 (SMNP Office report, 2020).

**Fig. 3:**
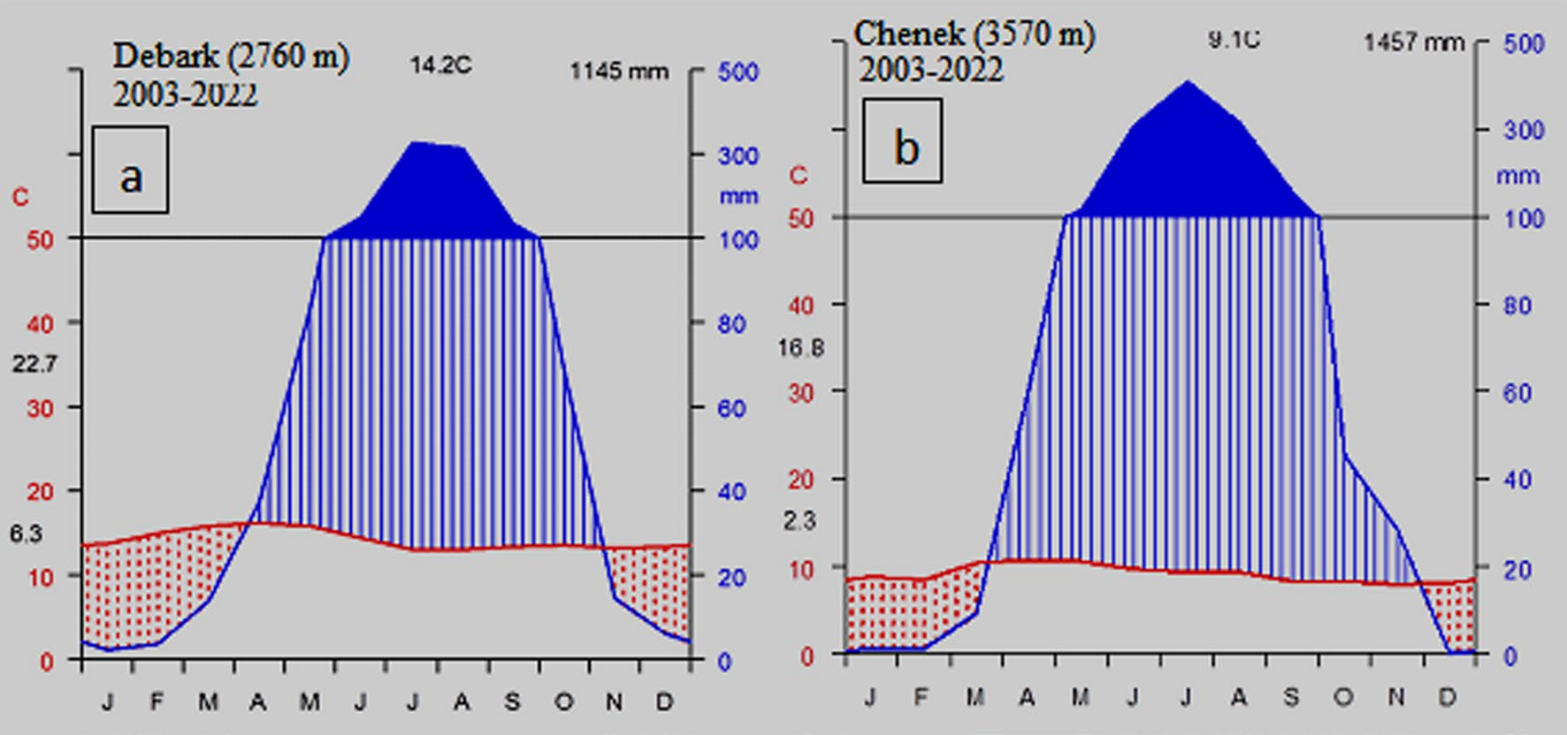
Climate diagrams for SMNP **a** for Debark and **b** for Chenek station

The climate of the Simien Mountains is quite different from the surrounding lowlands. Analyses of metrological data obtained from Ethiopian Meteorological Agency (EMA) Debark and Chenek stations (from 2003 to 2022) showed a unimodal rainfall pattern with variations in attitude. The mean annual rainfall for Debark and Chenek was 1145 and 1445, respectively. The area has low rainfall from October to March, which gradually increases to the peak period, June and August (Fig. 3). The mean annual temperature of Debark and Chenek was 14.2 and 9.1 °C, respectively. The coldest months are from October to January, whereas hotness occurs in April and May.

**Fig. 4:**
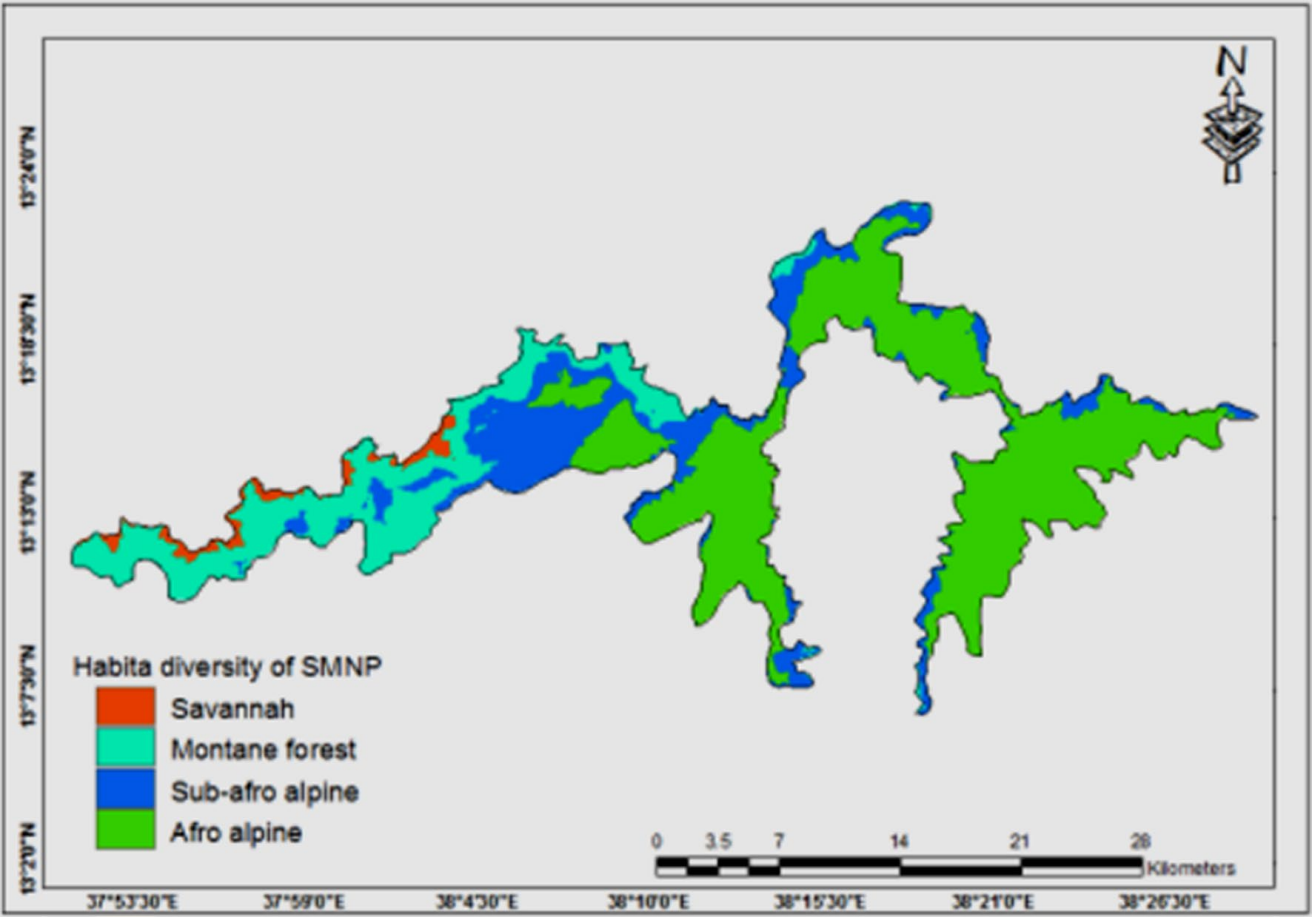
Vertical zonation of Simien Mountains’s vegetation

Vertically the vegetation of the mountains is classified into four. These are afroalpine, ericaceous belt, afromontane and woodland (Fig. 4). The afroalpine vegetation is largely made up of grasses and sedges covering the undulating plains, interspersed with *Lobelia rhynchopetalum,* and other herbaceous species. Small trees of *Hypencum revolutum* mixed with Ericas are sparsely distributed in less harsh areas. The ericaceous belt is a narrow zone between afromontane and afroalpine, ranging from 3000 to 3200 m altitude mostly occurs on the escarpment. The physiognomy of the *Erica* belt is largely characterized by the dominance of *Erica* species*, Hypericum revolutum* and perennial herbs such as *Thymus schimperi*. The afromontane vegetation is found in the area between 2,000 and 3,000 m a.s.l., in the less steep parts of the escarpment. Original afromontane forests are largely destroyed by human activities with the remaining patches confined to inaccessible areas [16]. Characteristic tree species in the forest patches include *Prunus africana* and *Olea eurpea* subsp. *cuspidata* with the prevalent climbers such as *Jasminum abyssinicum.* Moreover, the lowest areas contain savannah type wood and bushland.

### Study site and informant selection

**Fig. 5:**
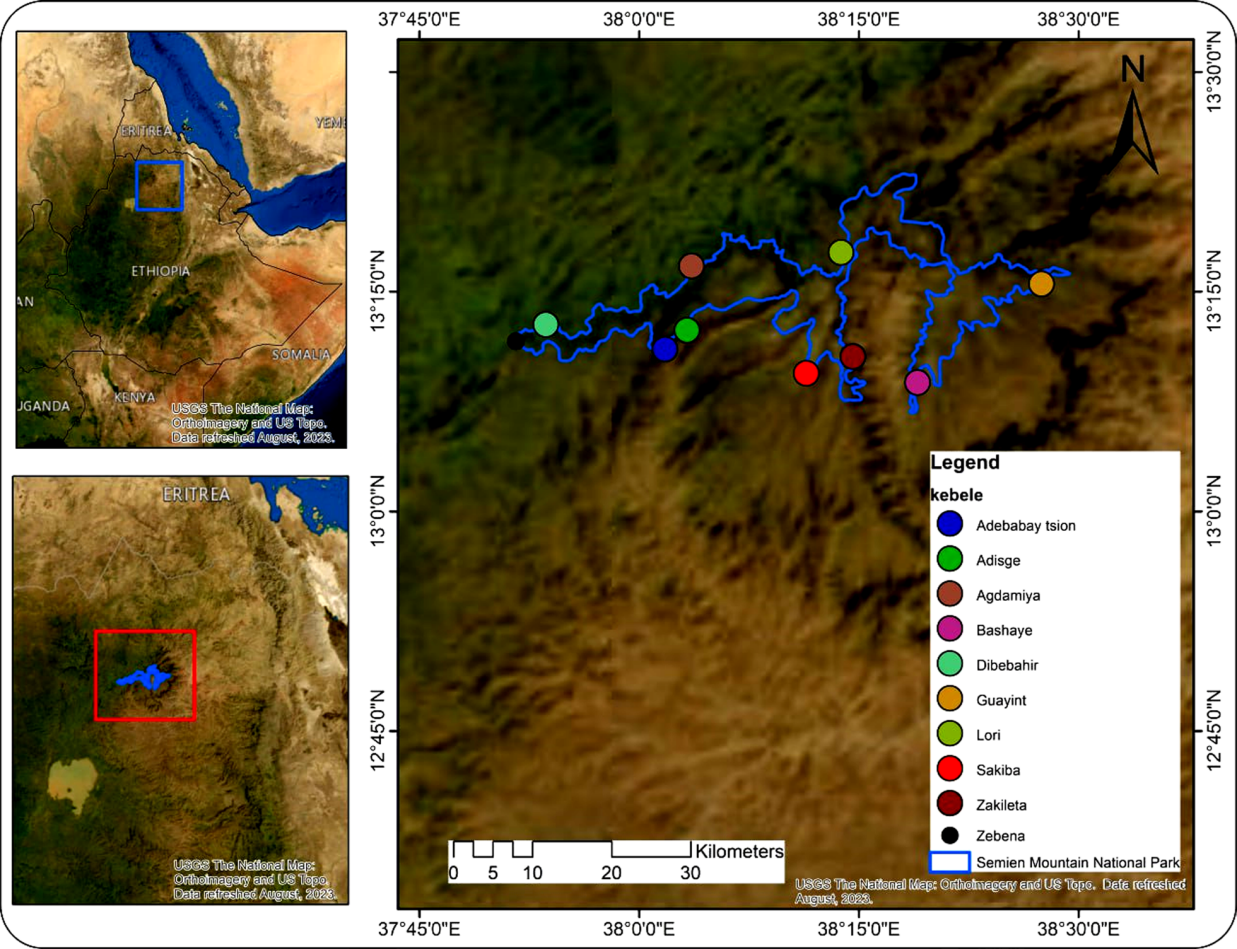
Map of Simien Mountains National Park with study sites neighboring the park

After conducting a reconnaissance survey, ten kebeles (the smallest administrative unit), among 42 buffering kebeles in the park, were selected for traditional lore data collection. Selection was made purposefully based on their relatively high plant diversity, altitudinal ranges, agroecology, traditional medicinal practices and the presence or absence of health facilities. Thus, as they met the selection criteria, Adebabay Tsion, Adisge, Bashaye, Agdamiya, Dibebahir, Guayint, Lon, Sakiba, Zakileta and Zebana were selected as study sites (kebeles) (Fig. 5).

A total of 100 informants (70 males and 30 females) aged between 20 and 90 years were selected from the sampled kebeles. Of these, 80 were selected randomly as general informants, and the rest 20 were selected purposefully as key informants based on the recommendations of local healers, elders and park rangers following [17]. Information on the study locations and informants’ demographic profiles are summarized properly (Table 1). Prior to commencing the interview, all participants were informed about the aim of the study. Following this, all informants voluntarily provided their oral consent. The ethnobotanical study was, then, conducted in accordance with the International Society of Ethnobiology (ISE) Code of Ethics 2006 (http://ethnobiology.net/code-of-ethics/).

**Table 1:** Location and demographic profile of the participants

Study kebele	Altitude	Longitude	Altitude	Ecology	No of HH	Gender		Age		Occupation		Religion	
						M	F	Yg	El	Lt	Il	Ch	Mu
Zakilta	421,118	1,452,694	2503	Highland	959	6	4	8	2	6	4	10	0
Sakiba	414,831	1,455,534	3609	Moorland	1285	7	3	6	4	4	6	10	0
Bashaye	426,293	1,452,091	3588	Moorland	1154	5	5	8	2	3	7	10	0
Guayint	446,569	1,462,748	3445	Moorland	650	8	2	4	6	4	6	10	0
Lori	415,321	1,469,242	3244	Moorland	955	6	4	2	8	3	7	9	1
Agidamiya	398,807	1,468,163	2483	Highland	1154	10	0	4	6	5	5	0	10
Adebaby	395,111	1,458,286	2411	Highland	1418	6	4	2	8	2	8	8	2
Adisgie	391,024	1,459,597	3138	Highland	796	6	4	4	6	5	5	3	7
Zebena	376,382	1,457,339	2889	Highland	559	9	1	2	8	2	8	10	0
Dib Bahir	383,553	1,463,149	1592	Midland	842	6	4	5	5	4	6	10	10

Key (NB): Occupation (Lt represents the interviewee who have completed primary school and has religious education & Illiterate, Il); House hold, HH; Age category (Young (20–39), Yg Elder, El); male, M, female, F; Religion (Christian, Ch, Muslim. Mu). Regarding to ethnicity and language all interviewees belong to Amhara ethnic group and speak Amharic language. Information on Gender, age, occupation & religion pertains to informants

### Traditional lore data collection

Data were collected through face-to-face or individual interviews, focus group discussions and guided field walks with. Field visits were done in different seasons with the aim of gathering medicinal plants during their flowering time. Interviews were conducted in Amharic language and run independently to make informants free to deal with secret information. During data collection, informants were communicated twice to validate the consistency and reliability of the information provided by them. Face-to-face interviews were used to collect secret or sensitive information that is considered personal, confidential, or culturally sensitive that could harm individuals, communities, or cultural traditions if disclosed without proper consent such as traditional healing practices, including medicinal plant use and its disclosure without proper authorization could be disrespectful. Information was gathered based on semi-structured questionnaires contained in a checklist that covered key topics regarding the informants’ profile, medicinal plants, parts used, method of remedy preparation, remedy dosage, route of remedy administration, antidotes or ingredients used, interaction of healers with the forest, ailments treated, marketability, threats and conservation practices of medicinal plants [17].

Focus group discussions (FGDs) were held by using 6–8 key informants selected purposefully from the different study sites, age groups and sexes. The discussions were used for ranking medicinal and other uses of medicinal plant species, enriching and confirming the reliability of the qualitative description of the information obtained during the face-to-face interview. Thus, it allows researchers to collect rich and in-depth qualitative data that helps to gain a deeper understanding of the complex relationships between people and plants within that specific cultural contexts, ultimately contributing to the conservation of traditional knowledge and the sustainable use of plant resources. On-site confirmations of the reliability of the information obtained during face-to-face interviews, with the help of practical demonstrations with informants, were also conducted through guided field walks. In addition, information regarding medicinal plant names, habits, habitats, status and market demand for medicinal plants was collected using a guided field walk with the informants. The medicinal plant specimen collections and photographs were taken during guided field walks. Local market surveys were carried out at three comparatively large market places in the study area: Debark, Qayit (Janamora) and Mekane-birhan (Janamora’s main city) local markets, so as to assess the marketability (selling and buying) of medicinal plants which is ultimately related to the survival of medicinal plants. Voucher specimen collections were done with the help of traditional healers. Specimens were air-dried, numbered, labeled, pressed, identified and deposited at the University of Gondar Herbarium. Identification of plant specimens was done both in the field and at the herbarium using floras of Ethiopia and Eritrea.

### Data analyses

Traditional lore variations and dynamics on the use of medicinal plants by males and females in different age groups and general and key informants were compared using a t test and one-way ANOVA (analysis of variance) at a 95% confidence level between means by using SPSS (Statistical Package for the Social Sciences) version 20. Basic traditional lore data such as plant habits and parts used, life form, source of medicinal plants, method of remedy preparation, route of remedy administration, ailments treated, threats and conservation practices of medicinal plants were analyzed via descriptive statistics such as percentage, frequency distribution and graphs as recommended by [17, 18].

In addition, ethnobotanical data clustering and ranking methods such as informant consensus factor (ICF), preference ranking, direct matrix ranking and fidelity level were carried out to ensure consistency and priority. ICF was computed for various categories of ailments in order to assess the level of agreement among informants regarding their knowledge of medicinal plants within each category. To determine ICF values, diseases were divided into nine groups, more or less, based on the international classification of diseases (ICD) [19], after which the ICF value was determined for each category. ICF values for nine disease categories were performed to understand knowledge homogeneity among informants for each disease category using the formula ICF = Nur-nt / Nur − 1 [20], where Nur = number of use citations in a particular ailment category and nt = number of medicinal plant species used for any ailment category.

A preference ranking exercise was conducted to evaluate the degree of preferences or levels of importance of eight selected medicinal plants used to treat bleeding using seven randomly selected key informants following [17]. Each informant assigned the highest value (7) for the most preferred plant species against bleeding and the lowest value (1) for the least preferred species. Finally, scores given for each plant species by the informants were summed and ranked. Such ranking helps to understand which plants, among the listed once, are considered more valuable to treat the specific disease mentioned, in this case bleeding. The use diversities of therapeutic plants were computed by direct matrix ranking (DMR). During the DMR exercise, seven widely utilized multipurpose therapeutic plant species were compared by selected key informants following [17]. The plants were listed by the selected key informants to assign use values to each species (5 = best, 4 = very good, 3 = good, 2 = less used, 1 = least used and 0 = not used). Accordingly, the scores assigned by key informants were added and ranked.

Fidelity level (FL) was used for species mentioned over 30 times to check their efficacy for a specific aliment following [21]. It was calculated as FL (%) = (Np/*N*) × 100, where Np is the number of informants that claim the use of a plant species to treat a particular disease and *N* is the number of informants that use the plants as a medicine to treat any disease, as stated by [22]. Fidelity level helps to measure the extent to which a plant is faithfully and consistently used for a specific use category, in this case treating a particular disease. Jaccard’s coefficient of similarity (Js) was used to compare the current study with other ethnobotanical studies in other parts of the nation. It was calculated using the formula Js = *c*/(*a* + *b* + *c*), where *a* represents the number of plants in an area "**a**," *b* represents the number of plants in area "**b**," and *c* represents the number of plants common to areas **a** and **b**.

## Results and discussion

### Relation of traditional lore and the natural environment

An independent sample test showed significant differences (*P* < 0.05) between traditional healers and general informants on the number of medicinal plant species they listed and associated use reports (Table 2). Similar results were reported by [23–25]. The significant difference observed between key and general informants could relate to the impact of age-old experience and the maximum degree of secrecy in using medicinal plants in the former and modernization in the latter. Based on their experience and assessing previous documents [23], told us community members who have greater contact with medicinal plants are more knowledgeable about the therapeutic uses of the plants than those with intermittent contact. Some informants even reported that several key informants are specialized for treating particular diseases like rabies and hepatitis (in local language it is called “YEWOFIE”).

**Table 2:** Statistical test of significance and independent t test on the number of medicinal plant mentioned by informants

Parameter	Informant group	*N*	Mean	*t*	*P* value
Gender	Male	70	15.13	− 4.729	0.058
	Female	30	10.40		
Informants	General informants	80	10.81	− 5.843	0.000**
	Key informants	20	25.30		
Age	Younger (20–39)	45	11.89	− 3.311	0.151
	Elder (40–90)	55	15.20		

∗Significant difference (*p* < 0.05); ** *t* (0.05) (two tailed), df = 98, *N* = number of respondents

The high level of indigenous knowledge contained in traditional practitioners may also be attributed to environmental advantages, cultural practices and public health services. Simien Mountains massifs, owing to their unique geological formation, wide altitudinal range and diverse topographic features, are endowed with a spectacular landscape. The combined effect of these factors gifted the mountain ecosystems with rich biodiversity, making the area part of the eastern afromontane biodiversity hotspot. SMNP, a portion of the massif, was declared as a World Natural Heritage Site by UNESCO in 1978 in honor of its unique combination of biodiversity and scenic beauty. The park covers a wide altitudinal range from below 2,000 m in the deep valleys to over 4,500 m a.s.l. in Ras Dashen (the highest peak in Ethiopia). Its topography, with gorges, crests, rocks and flat areas, results in a rich mosaic pattern of various habitats that favor the distribution of various species and increased isolation by impeding dispersal and pollination that ultimately result distinct species. The overall effect of these factors promotes to high species diversity with rich endemic elements. Thus, previous studies reported over 525 flowering plants [14, 16]. Therefore, higher species richness and diversity in the study area increase the chance of practitioners to prescribe the right remedy to their patients.

Christians, Muslims and Jews have lived in the SMNP and its environs for many generations, sharing strong cultural and economic ties. Jewish people have a strong connection to the environment and a tradition of using it for a variety of purposes, including the use of herbal medicine. As part of Israel's effort to gather its citizens from all over the world, the Jewish people have now largely been relocated from the area. However, their traditional wisdom and indigenous practices are left behind and become the wealth of local residents of the study area.

In the five districts sharing SMNP, there are 200,941 people and 54,576 households getting health services from three hospitals, 35 health centers and 100 posts. The most common public health issues that residents surrounding the park experiences include heart disease, hypertension, respiratory diseases, skin problems, injuries, diabetes, conjunctivitis and other eye infections (North Gondar Zone Health Office report, 2021). Access to infrastructure is limited in comparison with the national level. By deploying two female health extension workers in health posts at each kebele across the nation, government of Ethiopia launched disease prevention Health Extension Program. Nevertheless, the program did not bring the desired output in the primary health care of the SMNP residents. Female extension workers find it difficult to cross the undulating terrains of the dissected mountains’ topography to provide house-to-house health services. Thus, traditional healers, with a profound knowledge on local plants and diseases to be treated, are the only health workers immediately available to the inhabitants. In short, rich biological resources, strong cultural ties to the local resources, remote location and challenging topographic features for accessing modern health facilities make the settlers have a long tradition of using herbal traditional medicines as a primary health care.

Though males reported a higher number of therapeutic plants than females, the t test did not show a significant difference (*P* > 0.05) (Table 2). This finding is consistent with some other studies conducted in Ethiopia [24, 26–28], who claimed the absence of a significant relationship between gender and the number of medicinal plants listed by them. A higher number of plants reported by male informants than females, on average, could be related to the traditional flow of information along the male line in the nation [23]. On the contrary, this study disagrees with the previous reports by [25, 29] that highlighted a significant knowledge difference between the sexes. Besides, no significant difference (*P* > 0.05) was observed between the age groups (20–39 and 40–90 years) and the number of medicinal plant species they listed, as well as the respective uses they reported (Table 2). However, on average, elders mentioned greater number of medicinal plants and their uses (Table 2). This demonstrates that the therapeutic plants lore is still strong with elderly people [23] and lack of interest in acquiring the wisdom in young generation.

**Fig. 6:**
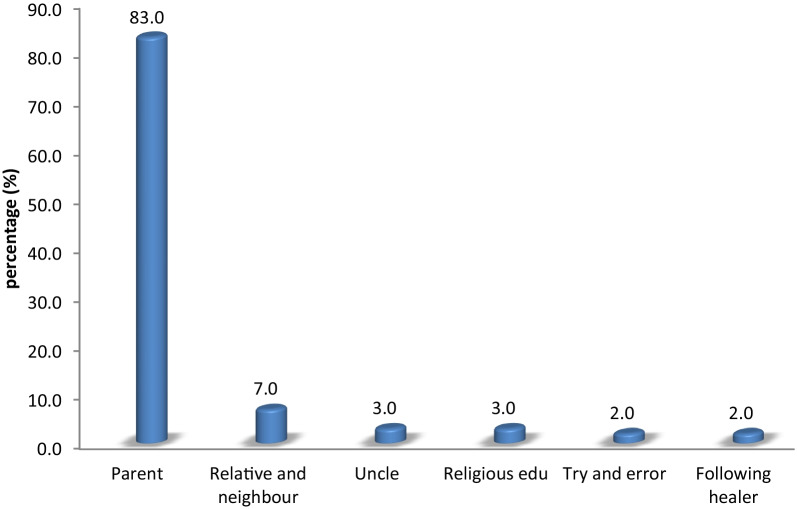
Ways of indigenous knowledge transfer

The present study highlighted the good knowledge base of residents on herbal remedy**.** Verbal communication, as reported by informants, was the most common way of transferring the traditional wisdom (83%) among the family members especially to the elder son. During transfer, secrecy is expected to be maintained in the family circle [23]. Wisdom obtained through other means was minimal (Fig. 6). In addition, some informants reported that they gained knowledge of medicinal plants by following traditional healers behind (without the permission of the healer) during medicinal plant harvest. Furthermore, a Muslim healer believed that their traditional wisdom was a gift from Allah or through religious learning.

The informants worried about the threat of indigenous knowledge as a result of younger generations reduced interest to learn about the medicinal plants due to the impact verbal transmission and modern education. Similar findings were also reported in other parts of Ethiopia [23, 30]. As a result, the absence of documented medicinal plants knowledge coupled with lack of interest in younger generation to learn indigenous wisdom threats future use of medicinal plants.

### Medicinal plants diversity in SMNP

A total of 113 medicinal plant species belonging to 92 genera and 56 families were documented. Except for one species, *Junperus procera*, all other medicinal plant species collected in the study area were angiosperms, with 90.3% being dicots and 9.7% monocots (Appendix 1). The species richness of medicinal plants in SMNP was higher than in Bale Mountains National Park [29], Mount Elgon, Kenya [31], Selale Mountain Ridges [27] and Gokand Valley, Pakistan [32], with 101, 107, 79 and 109 species, respectively. The higher numbers of medicinal plant species in SMNP might be associated with the mosaic pattern of microhabitats and great altitudinal variation (from below 2,000 m to over 4,500 m a.s.l.) that caused great ecological variation. In addition, the good number of therapeutic plants mentioned in the study area is another way of proving the rich traditional wisdom still practiced in the local community for their health care. Local communities adhere to the ancestral medical traditions by upholding them as a highly valued heritage of society [23].

**Fig. 7:**
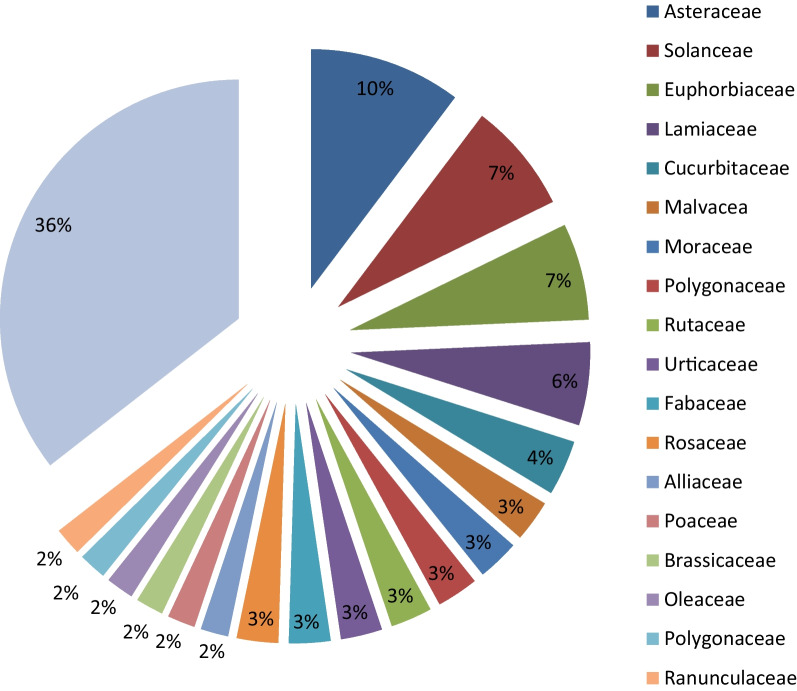
Families of medicinal plants in the study area

Asteraceae was the most therapeutic plant species-rich family (10%), followed by Solanaceae (7%), Euphorbiaceae (7%), Lamiaceae (6%) and Cucurbitaceae (4%). There were seven families represented by three species, six families were represented by two species, and the remaining 38 families were represented by one species. The aforementioned eighteen families accounted for about 64% of all species. Euphorbia was a species-rich genus (4 species), followed by *Ficus*, *Remex,* and *Solanum,* represented by three species each. Three genera (*Alium*, *Echinops,* and *Helichrysum*) were represented by two species each, and all other genera (89) were represented by one species each (Fig. 7, Appendix 1). The dominance of Asteraceae was also reported in [23] (12 species), [33] (19 species), [34] (25 species) and [32] (six species). Its dominance may be explained by species abundance, which is linked with their efficient dispersal mechanisms at higher altitudes in high mountains. The wide application of species from these families might also be associated with the presence of effective bioactive chemicals against ailments.

**Fig. 8:**
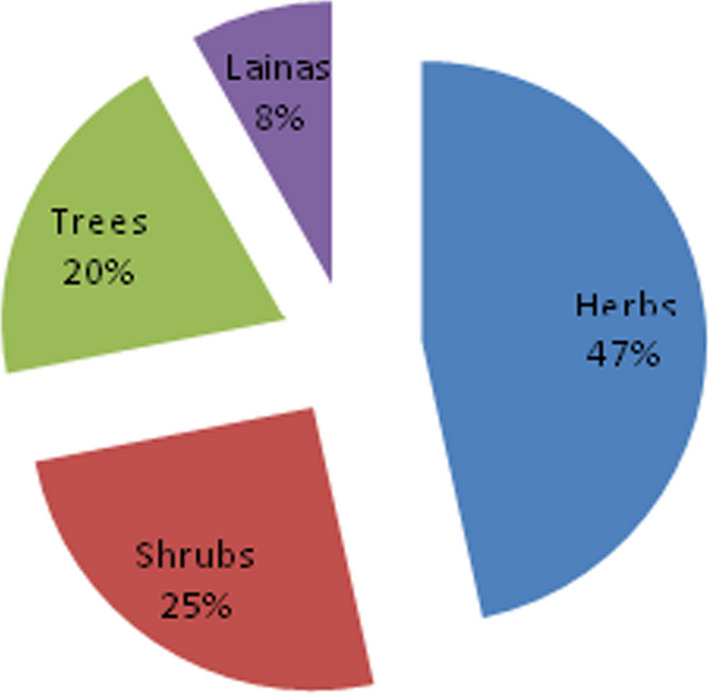
Growth habit of medicinal plants

**Fig. 9:**
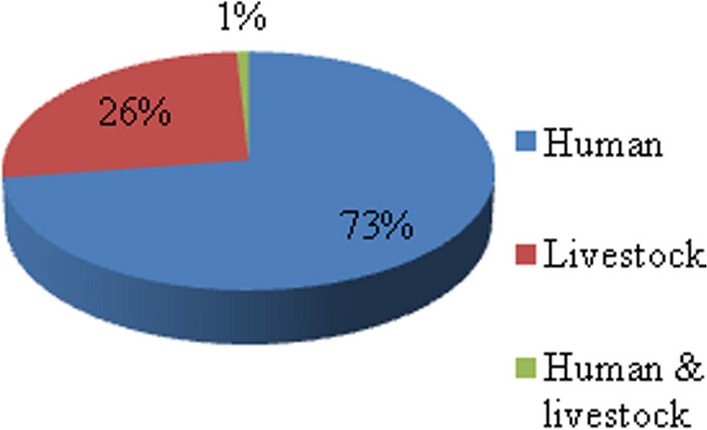
Plant species against ailment types

In terms of plant growth habit, 36 families, 47 genera and fifty-three species were herbs; twenty families, 26 genera and 28 species were shrubs; 15 families, 18 genera and 22 species were trees; nine families, nine genera and none species were lianas (Table 3). Therefore, most of the therapeutic species recorded were herbs (47%), followed by shrubs (25%) (Fig. 8). The dominance of herbs was also disclosed by [29] (54.46%), [34] (47%) and [32] (52.29%). Their abundance might be due to their abundance at higher altitudes since vegetation stature decreases as altitude increases, which ultimately leads to a higher abundance of herbaceous species with different growth forms (forbs, grasses, giant herbs, etc.). In addition, the ease of availability in the nearby area and the efficacy of herbs made local people depend more on herbs [33]. Conversely, the findings of this work were contradictory to some other previous works [35, 36], who reported shrubs were the dominant medicinal species due to their year-round harvest and their relative tolerance to any form of disturbance. The therapeutic plants collected in the study area were reported to treat 87 different aliments, of which 73% were known to treat human ailments only (Fig. 9). Similar results were reported in previous works [35, 37]. This demonstrates that greater emphasis is given to treating human health problems as compared to livestock [35].

**Table 3:** Classification of medicinal plants

Category	Sub category	Growth habit	No of families	No of genera	No of species	Total species (%)
Angiosperms	Dicots	Herbs	29	39	44	38.94
		Shrubs	20	26	28	24.78
		Trees	14	17	21	18.58
		Lianas	8	8	8	7.08
	Monocots	Herbs	7	8	9	7.96
		Shrubs	–	–	–	–
		Trees	–	–	–	–
		Lianas	1	1	1	0.88
Gymnosperms	Trees	1	1	1	0.88	
	Shrubs	–	–	-	-

Among the recorded medicinal plants in SMNP, thirteen (11%) of them were endemic to Ethiopia. These were *Cynoglossum coeruleum* subsp. *coeruleum. Verbascum stelurum*, *Echinops kebericho*, *Inula confertifora*, *Lobelia rhynchopetalum*, *Urtica simensis*, *Aloe steudneri*, *Helichrysum horridum*, *Vernonia rueppellii*, *Thymus schimperi*, *Kniphofia foliosa*, *Kalanchoe petitiana,* and *Impatiens tinctoria.* From these endemic plants, *Urtica simensis*, *Aloe steudneri*, *Helichrysum horridum*, *Vernonia rueppellii*, *Thymus schimperi*, *Kniphofia foliosa*, *Kalanchoe petitiana,* and *Impatiens tinctoria* were included in the IUCN (International Union for Conservation of Nature) Red Lists [38]. *Kalanchoe petitiana* and *Echinops kebericho* were also reported by local people as locally threatened species in the area (Table 6). Being unique to Ethiopia, further analysis of their potential medical benefits could provide novel and significant scientific findings.

### Sources of medicinal plants

**Fig. 10:**
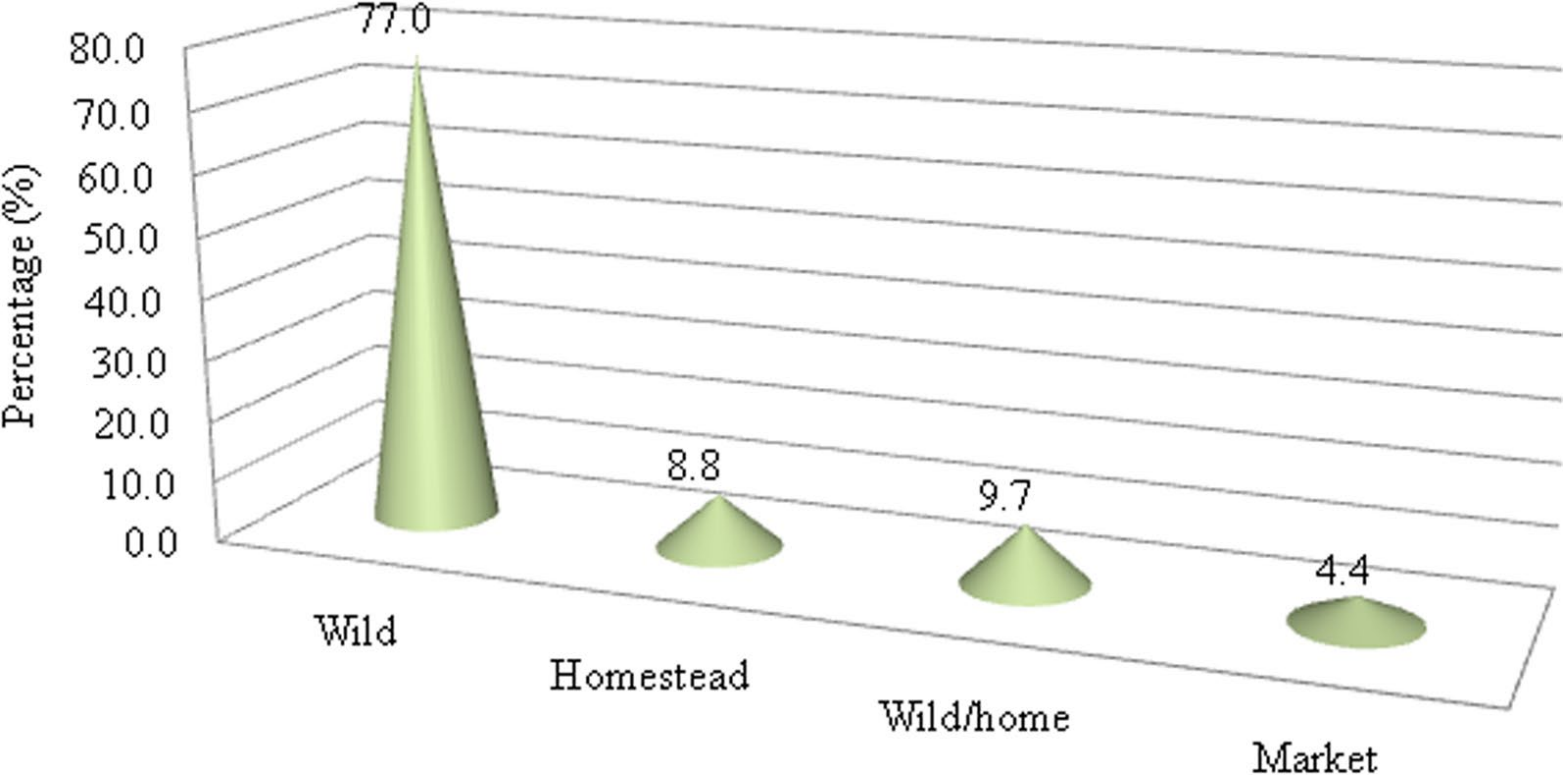
Sources of medicinal plants

The major source of medicinal plant species was the wild natural vegetation (77%). Some therapeutic plants were collected both in wild and cultivated areas (9.7%) and homesteads (8.8%) as well as few of the plants, such as *Zingiber officinale, Citrus aurantiifolia,* and *Coffea arabica,* were found in markets (4.4%) (Fig. 10). The result of this work agreed with similar previous works [24, 36, 39]. This implies that natural vegetation still contains a good number of therapeutic plants, or that local communities do have little practice of conserving medicinal plants in their homesteads. Thus, a good opportunity for the inhabitant’s bordering SMNP to access medicinal plants from the park might make the natural vegetation the main source of medicinal plants.

### Plant parts used in the study area

**Fig. 11:**
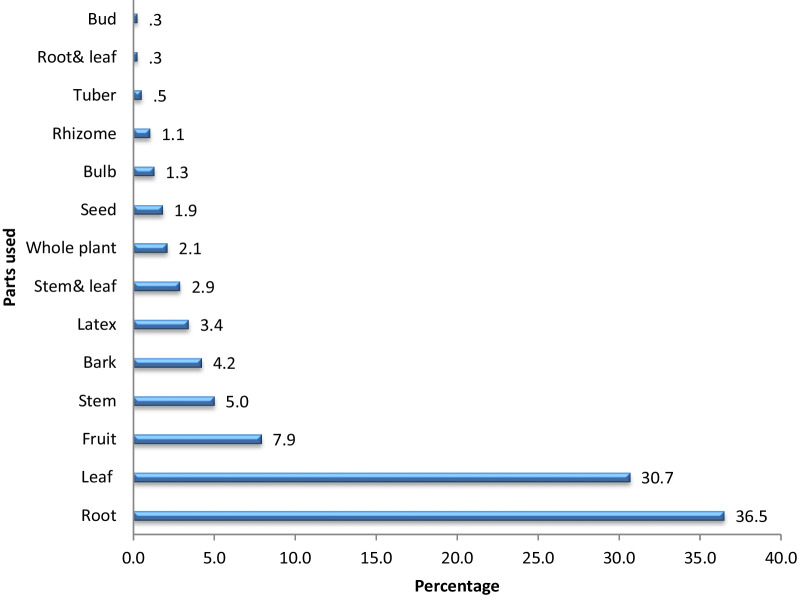
Plants parts used for remedies (%)

Roots were the most widely used plant part (37%), followed by leaves (31%). Other plant parts that were utilized to prepare remedies made about 0.3–7.9% of the total (Fig. 11). The dominance of roots for remedy preparation was also reported by other similar previous studies [29, 31]. The local people perceived that roots are available in all seasons and that active biological ingredients are more common at the tips of roots and leaves. A similar result was also disclosed by [40], who reported that the presence of more antibiotic chemicals in roots than other plant parts makes roots the most preferred. Conversely, most previous studies, including [34, 35], reported that leaves are the most commonly used plant part for remedy preparation since, unlike roots, harvesting leaves does not have detrimental effects on the survival of medicinal plants. In addition, secondary metabolites are largely produced and concentrated in leaves.

### Conditions of remedy preparation, preparation methods and administration routs

**Fig. 12:**
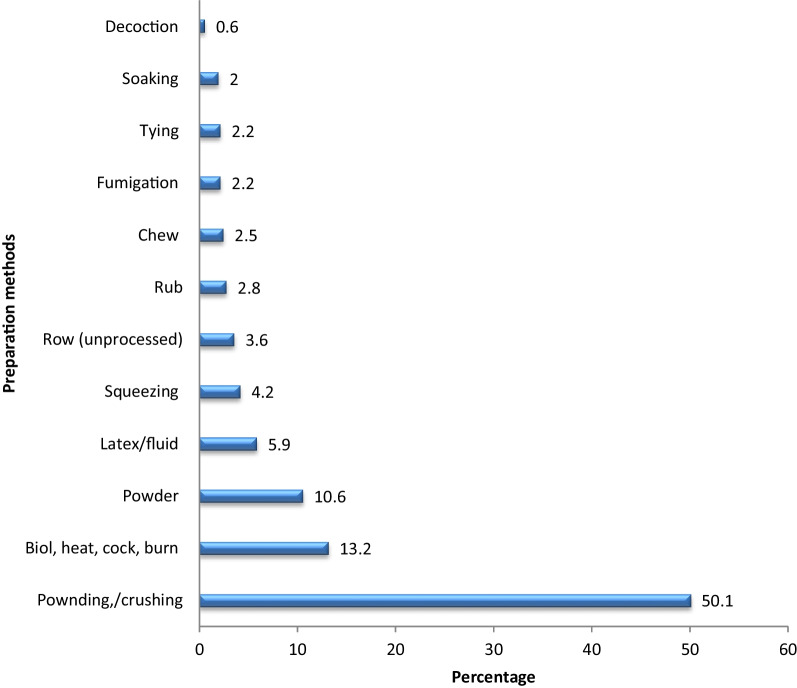
Remedy preparation methods

Most of the remedies were prepared from fresh plant parts (66.6%), whereas 17.5% and 15.9% were prepared from dry and fresh/dry forms, respectively. The result was consistent with the findings of the previous studies [28, 41, 42], who reported that the use of fresh forms increases the efficacy of medicinal plants since the secondary metabolites that have antimicrobial activities are retained in the extract since the chemicals are easily removed when they dry up. The remedies were commonly prepared by pounding (50.1%). Other preparation methods were far less common than pounding, which accounted for 13.2% of boiling (heating) to 0.6% of decoction (Fig. 12). This result is consistent with similar previous works [23, 43, 44]. The reason pounding is most frequently employed is the assumption that pounding releases the therapeutic ingredients into the extracts through heavy, repeated striking of medicinal plants during the procedure.

**Fig. 13:**
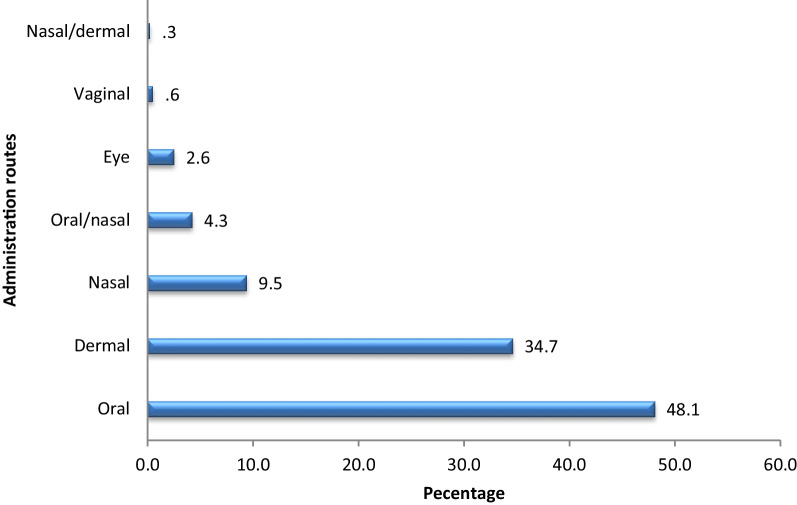
Routes of remedy administration

The most frequently used route of remedy administration was oral (48.1%), where the remedies are taken by drinking or eating, followed by dermal, where remedies are creamed, rubbed, or bathed (34.7%). Other routs of were far less frequent than oral and dermal, which accounted for 9.5% of nasal and 0.3% of nasal/dermal cases (Fig. 13). Similar reports have been made by [35, 36, 42]. Oral rout is preferred due to the rapid reaction of the medicine to treat the illness that the local healers realized through their longer experience [36].

### Ethnobotanical data scoring and ranking

#### Efficacy of medicinal plants

The highest ICF value (84%) had been shown for categories of respiratory disease and febrile illness. On the other hand, the category of central nervous system (CNS)-related diseases had the lowest ICF value (57%). The highest plant use citation was also found for the same disease category (Table 4). The highest ICF values demonstrated greater agreement among respondents’ on the use of therapeutic plant species reported for treating respiratory disease and febrile illness. The observed highest informants’ agreement, coupled with high plant use citations for the disease category, could also confirm the relatively higher incidence of the diseases in SMNP. According to [45], when information is exchanged between informants, ICF approaches to one demonstrating higher information exchanges between the communities living around SMNP.

**Table 4:** Informant consensus factor by categories of diseases in SMNP

Disease category	Aliment\disease	Number of plant species (Nt)	Number of use citation (Nur)	ICF (%)
Respiratory & febrile illness	Tonsillitis, Common cold, cough, Tuberculosis, MICH, Nasal discharge	28	171	0.84
Trauma & Musculoskeletal	Dislocation, Fracture, scorpion & Snake bite, beating with stick (Yebetir), Burn & Arthritis	21	92	0.78
Hemorrhoid, wound & swelling	Wound & bleeding, Swelling & Hemorrhoid & Ganglion cyst	23	93	0.76
Eye	Eye cellulitis, Chalazion & vision impairment	10	36	0.74
Gastrointestinal & Hepatic	Abdominal distention, Abdominal pain, Constipation, Rectal prolapse, Gastritis, Gastroenteritis, Toothache, Appetite & Hepatitis	33	102	0.68
Helminthiasis	Ascariasis, Tape worm, leeches	20	53	0.63
Dermal	Atopic Dermatitis, Eczema, Boils, Tinea corporis, Tinea Unguniunm, Tinea versicolor, Athletfoot, Dandruff, lice & Skin fissure (chefachft)	23	55	0.59
Genitourinary & Reproductive	Bedwetting, Circumcision, Urinary retention, syphilis, Retained placenta, Uterine myoma, Impotence, infertility, Abortion and bleeding after delivery	29	68	0.58
CNS (Neurological & Psychiatry)	Headache, Epilepsy, Hypertension, evil spirits, evil eye & Rabies	33	77	0.57

#### Priority ranking of therapeutic plants

The preference ranking exercise was used to identify the most preferred medicinal plants from the list of plants that stop bleeding in the study. Thus, the ranking score disclosed that *Rumex nepalensis* stood first, showing the most preferred species for treating bleeding, followed by *Achyranthes aspera* (Table 5). On the other hand, *Ficus palmata* was seen to score the least preference value relative to other species to stop bleeding*.* Other therapeutic plant species listed in the priority ranking *(Malva verticillata, Plantago lanceolata, Rumex nervosus, Aloe steudneri,* and *Datura stramonium)* had medicinal value in between the first and least ranked therapeutic plant species to stop bleeding. This finding disagrees with the finding of [24], who reported that *Achyranthes aspera* was the most preferred plant to stop bleeding. On the other hand, the fact that *Achyranthes aspera* has been mentioned as a medicinal plant in other study area may indicate that the species possesses medicinal properties that have been used to halt bleeding as well as the dissemination (or exchange) of indigenous knowledge among communities across different geographical areas.

**Table 5:** Preference ranking values of eight medicinal plants used to stop bleeding

Medicinal plants	Respondents (R1–R7)								
	R1	R2	R3	R4	R5	R6	R7	Total	Rank
*Achyranthes aspera*	4	3	4	4	5	5	4	29	2nd
*Aloe steudneri*	5	3	2	2	3	1	2	18	6th
*Datura stramonium*	3	1	3	2	2	1	2	14	7th
*Ficus palmata*	1	1	2	2	2	0	2	10	8th
*Malva verticillata*	4	3	5	4	3	4	3	26	3rd
*Plantago lanceolata*	2	3	4	3	3	3	5	23	4th
*Rumex nepalensis*	5	4	5	4	5	4	4	31	1st
*Rumex nervosus*	3	4	2	3	2	2	3	19	5th

#### Use diversity of medicinal plants

A direct matrix ranking exercise was used to test the use diversity of multipurpose therapeutic plants in the study area. This was also used as a means to identify which of the multipurpose plant species is under severe anthropogenic pressure with the corresponding threatening factor. Accordingly, *Olea europaea* was the most desired multipurpose plant species, followed by *Hagenia abyssinica,* whereas *Brucea antidysenterica* was the least wanted multipurpose plant species. In other words, *Olea europaea* and *Hagenia abyssinica*, the most desired multipurpose therapeutic plant species, become endangered due to overharvesting (Table 6).

**Table 6:** Average direct matrix ranking scores of eight key informants for seven species

Medicinal plant	Use categories						
	Med	FD	Con	forg	Agr tool	Total	Rank
*Brucea antidysenterica*	3	1	2	0	1	7	7th
*Carissa spinarum*	3	2	1	1	2	9	5th
*Dodonaea angustifolia*	3	1	2	1	1	8	6th
*Hagenia abyssinica*	5	4	0	0	4	13	2nd
*Lobelia rhynchopetalum*	4	3	1	3	0	11	3rd
*Olea europaeasubsp. cuspidata*	3	4	3	1	3	14	1st
*Rumex nepalensis*	5	1	0	4	0	10	4th
*Total* *Rank*	26 1st	16 2nd	9 5th	10 4th	11 3rd		

Key: Med = medicinal, FD = firewood, Con = construction, Forg = forage, Agr tool = agricultural tool

The result could tell us these plants are exploited for several household purposes. Overharvesting of multipurpose medicinal plant species for medicinal use, agricultural tools and firewood purposes was the major factor in the decline of the species in SMNP. Previous authors [23, 29, 46] also disclosed that overharvesting of multipurpose medicinal plants for different purposes fastens the depletion of plant species in different regions of Ethiopia. According to [23], a plant's use diversity indicates how frequently it is used, which contributes to its decline in the area. These authors, based on the results of their work, called for urgent complementary conservation action (*in-situ* and *ex-situ*) to save the fast-eroding multipurpose medicinal plant species.

#### Fidelity level (FL)

It helps to measure the extent of faithful and consistent relative healing potential of each species treating a particular disease. In SMNP, the highest fidelity level was recorded for *Rumex nepalensis* (92%) for treating wound, followed by *Phytolacca dodecandra* (90%) for treating rabies (Table 7). A higher fidelity level is considered as a clue for the high healing potential of these plants against the corresponding diseases. Secondary metabolites with wound healing effects were previously reported from *Rumex nepalensis* by [47, 48]. This might be the reason why local people use the species widely to treat wounds. Medicinal plants with the highest fidelity level indicate that they are widely used by the local people [20]. In the study conducted by [36], *Phytolacca dodecandra* was found to have the highest fidelity level for treating rabies. The use of the same plant species for the same ailment in different localities demonstrates their cosmopolitan distribution and the fact that such plant species are effective for treatment of specific ailments. On the other hand, *Echinops kebericho* was seen scoring the lowest FL value (46%) for treating the evil eye (Table 7). The highest fidelity value indicated the most faithfulness of species to treat a given aliment and vice versa [3]. Plants with the highest ranking value, ICF and/or fidelity level could be candidates for further phytochemical investigation to prove the bioactive components responsible for their high healing potential [23].

**Table 7:** Fidelity level of the medicinal plant species used to treat the most common ailments

No	Medicinal plant	Disease type	Np	*N*	FL	FL%	Rank
1	*Clutia abyssinica*	Tinea corporis	7	14	0.5	50	7th
2	*Echinops kebericho*	Evil eye	13	28	0.46	46	8th
3	*Jasminum abyssinicum*	Toothache	8	13	0.61	61	6th
4	*Phytolacca dodecandra*	Rabies	9	10	0.9	90	2nd
5	*Rubia cordifolia*	Cough, common cold	10	13	0.76	76	4th
6	*Rumex nepalensis*	Bleeding, wound	24	26	0.92	92	1st
7	*Verbasicumstelurum*	Abdominal pain	12	18	0.66	66	5th
8	*Zehneria* *scabra*	Febrile illness	26	31	0.84	84	3rd

### Marketability of medicinal plants

Local market surveys revealed that some therapeutic plants, *Rubia cordifolia* and *Securidaca longepedunculata,* were sold in the market for the treatment of cough and evil eye, respectively. While in the remaining local markets, plants were sold for other purposes such as food (*Lycopersicum esculentum, Allium sativum, Allium cepa)*, fumigants for a pleasant smell at home (*Cymbopogon citrates, Silene macrosolen, Olea europea* subsp *cuspidata, Otostegia integrifolia*)*,* tooth brushes (*Olinia rochetiana, olea europea* subsp *cuspidata),* and additives to fermented beverages (Tella) (*Rhamnus prinoides*) and spice (*Ruta chalepensis*) The less marketability of medicinal plants was also reported by [30]. Most of the time, healers prescribe medication to patients at their home rather than bringing medicinal plants into the market with the intention of making money.

Though most of the medicinal plants are not found in the market for the medicinal purposes, it was observed that they are marketable for other purpose. The marketability of medicinal plants, for any purpose, is closely associated to the conservation risk of the plant species. Market demand for medicinal plants (either for medicinal or others uses) can put pressure on wild populations that may lead to overharvesting and unsustainable collection practices that pose a conservation risk. The marketability of medicinal plants can also drive illegal trade by targeting species of high commercial value. In response to market demand, unsustainable harvesting practices may be employed, such as collecting plants at an immature stage or removing the entire plant instead of selectively harvesting specific parts. These practices hinder regeneration, disrupt ecological interactions and affect the long-term survival of the species. Promoting sustainable cultivation and domestication of medicinal plants can help reduce the reliance on wild collection and alleviate the conservation risk associated with high market demand.

### Jaccard’s coefficient of similarity

Jaccard's similarity coefficient was performed to compare the therapeutic plant species composition similarity with seven other previously studied areas in the nation. It has been found that the SMNP shared the highest similarity (34.53%) with Yilmana Densa and Quarit Districts, followed by Ada’a District (31.72%). This similarity might be due to shared common culture and religion. Though SMNP and Bale Mountains National Park are similar ecosystems, they share a lesser therapeutic plant species similarity (9.14%). This may be the result of differences in the people’s historical and cultural traditions. The study conducted in the Erer Valley of Babile Districts shared the least number of common therapeutic plant species (3.77%) with SMNP (Table 8). This may be due to the altitudinal range differences between the two areas (Table 8). The elevation ranges of Erer Valley in Babile lie between 940 and 1585 m, while SMNP lies between the ranges of 1500 and 4500 m above sea level. Variations and similarities in cultural practices and environmental factors, as well as the proximity of regions, are the causes behind the level of exchange of traditional knowledge and medicinal plants across various locales.

**Table 8:** Jaccard’s coefficient of similarity (Js) between the current study and other similar studies conducted in Ethiopia

Sample of study areas	A	B	C	JCS (%)	References
Simien Mountains National Park	114	–	–	–	Present
Bale Mountain National Park	101	83	18	9.14	[29]
Ensaro District, North Shewa zone	44	18	26	19.7	[46]
Erer Valley of Babile Districts	51	45	6	3.77	[49]
Yilmana Densa and Quarit Districts	112	54	58	34.53	[25]
Enarj Enawga District	111	59	52	30.05	[41]
Ada’a District, East Shewa	131	72	59	31.72	[26]
Dalle District, Sidama	71	45	26	16.35	[50]

### Threats and conservation of medicinal plants in the study area

Associated with the rapid population growth, the demand for agricultural and grazing land is increasing. Consequently, the vegetation of the study area is shrinking, followed by a decline in the availability of therapeutic plants over time. A preference ranking of six factors that threaten medicinal plants showed overgrazing as the most serious threat, followed by agricultural expansion. Therapeutic plant collection was ranked as the least threatening factor (Table 9). After the relocation of villages (Gich and Arkwazeye) from the park and removing the road out of the park boundary, overgrazing and agricultural expansion are identified as the major factors threatening the biodiversity of the park (SMNP Office report, 2020). This finding is in line with the study conducted in Gozamin district [29]. On the other hand, the studies conducted in other areas of the nation [49–51] reported agricultural expansions as the principal threatening factor. Further analysis showed that *Echinops kebericho* was found to be the most threatened and rare therapeutic plant species, followed by *Kalanchoe petitiana* (Table 10). Similarly, the same species is reported as rare in the Sheka Zone [52]. Frequent use of its root as traditional medicine might be the reason for its current rare occurrence.

**Table 9:** Presence ranking of threatening factors to TMPs

Threatening factor	Respondents									
	R1	R2	R3	R4	R5	R6	R7	R8	Total	Rank
Agricultural expansion	4	4	4	5	4	3	4	3	31	2nd
Overgrazing	3	4	5	4	5	5	4	5	35	1st
Fire (intentional)	5	3	3	2	4	3	4	4	28	3rd
Firewood and charcoal	3	4	3	2	3	4	3	2	24	4th
Medicinal	2	2	1	3	1	2	1	2	14	6th
Drought	2	3	1	3	2	3	3	2	19	5th

**Table 10:** Priority ranking of selected TMPs based on their degree of scarcity in the wild

Threatened medicinal plant	Respondent									
	R1	R2	R3	R4	R5	R6	R7	R8	Total	Rank
*Brucea antidysenterica*	4	3	2	1	3	3	3	2	21	5th
*Echinops kebericho*	5	5	4	5	4	3	4	5	35	1st
*Kalanchoe petitiana*	5	4	3	5	4	3	5	4	33	2nd
*Rumex abyssinicus*	3	4	3	3	2	5	4	3	27	3rd
*Silene macrosolen*	3	2	5	3	3	4	4	1	25	4th

Regarding conservation practices, 89% of informants said that local communities exert little effort and do have a weak tradition of conserving therapeutic plant species. Few members of the community attempt to grow plants on their homesteads for therapeutic purposes. Similar findings were also reported in Ankober district [23]. The reason not to attempt growing in their homesteads might be related to the fact that they still have an opportunity to collect therapeutic plants in the park.

### Novel ethnobotanical findings

New insights were revealed by a comparative analysis of the present work and earlier ethnobotanical research conducted in Ethiopia. It was reported for the first time that *Helichrysum horridum* (endemic to Ethiopia), *Helichrysum citrispinum,* and *Vernonia rueppellii* had traditional therapeutic values. Pounded roots of *Helichrysum horridum* and leaves of *Inula confertiflora* with butter were reported to treat cattle eye disease. Likewise, its dried and pounded root, together with the seeds of *Lobelia rhynchopetalum* and *Sesamum indicum,* was recorded to treat uterine myoma. In addition, tying its internal stem bark around a child’s waist is used to avoid child bed waiting. Eating the crushed roots of *Helichrysum citrispinum* was reported for the treatment of a disease locally called “MICH” (a fetal disease caused by sitting on warm, moist soil or by exposing to suffocated material during sunny time). *Vernonia rueppellii* fresh leaves were reported to stop bleeding*.* Even this species was not mentioned in the globally reviewed 109 medicinal species of the genus *Vernonia* [53]. *Aloe steudneri* (endemic to Ethiopia) was also the first to be reported for the treatment of human wound. These include bleeding, wounds and snake bites. In addition, it is used to make hair black and smooth. Moreover, it was reported to make cattle and sheep more muscular. This might be related to the clearing of intestinal parasites, as [54] reported.

Even though *Dodonea angustifolia* was reported for the treatment of different ailments by different authors, it is the first to be mentioned against Corona Virus Disease 19 (COVID-19). This study is also the first to report *Kosteletzkya begoniifolia* against abortion and scorpion bite. But it was reported for the treatment of anthrax [55] and body swelling [55, 56]. Even though *Haplocarpha schimperi* was reported for different medicinal values [23, 34, 57], the current study recorded leaves for the treatment of Tinea versicolor, which is the first report in Ethiopia. *Lobelia* *rhynchopetalum* and *Satureja simensis* are also mentioned only in this study against epidemic diseases and baby weight loss, respectively. The root of *Carduus schimperi* and the bark of *Ficus vasta* are also used for the treatment of sheep nasal discharge and female infertility, respectively, which is the first documentation in Ethiopia.

The specific medicinal use(s), either in single or in combination, of *Helichrysum horridum*, *Inula confertiflora, Lobelia rhynchopetalum*, *Sesamum indicum*, *Aloe steudneri*, *Satureja simensis, Carduus schimperi, Haplocarpha schimperi* and *Kosteletzkya begoniifolia* described in this work are also novel or findings reported for the first time in Africa. These medicinal plants are all endemic to Ethiopia.

## Conclusion

The higher therapeutic plant species richness of the study area demonstrates that SMNP is serving as an *in-situ* conservation site and refuge for many endemic and endangered species. In other words, the documentation of a higher number of therapeutic plants proves the rich traditional lore of the local community in using therapeutic plants for their health care is still continuing. Increased demand for free grazing and agricultural expansion highly threatens the vegetation of the study area in general and therapeutic plants in particular. Erosion of therapeutic plants, verbal transfer via family line, great secrecy and lack of interest of the young generation in acquiring the traditional lore of therapeutic plants led to the deterioration of the long tradition of using therapeutic plants for health care. Practitioners should be encouraged (through various means) to share and bring their traditional wisdom to the scientific frontage, and highly preferred and endangered therapeutic plant species like *Echinops kebericho* should* g*et priority for conservation measures, and local healers need to grow such plants in their homesteads.

## Data Availability

All the necessary data collected for this study were analyzed and included in the tables, figures and appendix of the manuscript.

## Appendix 1: Medicinal plants, their habit, parts used, ailments treated, habitat, method of preparation and administration

**Table:**

Scientific name (Family, Local Name)	Classification	Ht	Intended use	Ra	Ailment treated	PPU, CP and methods of preparation	Plant source	Specimen code	GPS
*Cynoglossum coeruleum* Hochst. ex. A.DC. (Boraginaceae, Chegegot)	Di	H H	Hum	D	Febrile illness	Rub fresh leaves and applied topically	W	E001	X 395839
			Hum	D	Dysentery	Fingertip size of fresh roots pounded; mixed with cup of coffee and then drunk			Y 1465037
			Hum	Op	Eye cellulitis	Fresh leaves pounded and applied on eye			
*Artemisia abyssinica* Schtz. Bip. ex Rich (Asteraceae, Chikugn)	Di		Hum	N	Common cold	Fresh leaf and stem placed in nostril	W	E002	X 394434 Y 1462614
			Hum	N/O	Evil eye	Fresh roots pounded combined with roots of *Ferula communis* and *Verbasicumstelurum* mix water and drunk or wrapped with a piece of cloth and dampen; then place in nose to smell			
			Hum	D	Febrile illness	Crushed dried leaves and stem together with *Thymusschimperi* Ronniger, and smeared on skin			
*Asparagus africanus* L. (Asparagaceae, Yeset–kest)	Mo	H	Hum	D	Burnt body	Pounded the fresh leaves and applied on injured areas for 2 days	W	E003	X398797 Y 1463416
*Bersama abyssinica* Fresen (Melianthaceae, Azamir)	Di	T	Hum	O	Gastric enteritis (Tsidaki)	Powdered the fresh leaves and mixed with water, then taken orally for 7 days	W	E004	X 377597 Y 1458471
*Buddleia polystachya* Fresen. (Buddlejaceae, Anfar)	Di	T	Hum	D	Bleeding after delivery	Fresh leaves cut by knife and buried outside door on fixed stone by calling her Christ name	W	E005	X 396015 Y 1462675
			Hum		To make TELLA	Fresh leaves crushed and wash the containers of “Tella” local beer			
					Washing cloth	Fresh leaves crushed, pounded and used as “traditional” soap			
*Calpurnia aurea* (Ait.) Benth (Fabaceae, Zigta)	Di	Sh	Hum	O	Hepatitis	Pounded fresh/dried leaves and seed together and mixed with water, then 1/3 of a cup is drunk and eat boiled lentil after taking medication	W	E006	X 394308 Y 1459262
			Lk	D	External parasite	Fresh leaves pounded and mixed with water; then applied on affected area			
			Hum		Wound	Fresh leaves pounded and mixed with saliva then applied on wound			
*Carissa spinarum* L. (Apocynaceae, Agam)	Di	Sh	Hum	N	Evil eye	Fresh/dried roots pounded together with roots of *Lobelia rhynchopetalum*, Thymus *schimperi, Verbasicumstelurum and bulbs of Allium sativum* then powdered and wrapped by piece of cloth and place in noses	W	E007	X 401941 Y 1469114
			Lk	N	Mich	Dried roots pounded and heated on fire and fumigate			
			Lk	O	Rabies (for Dog itself)	Powdered fresh roots placed inside bread and given to eat			
*Clematis simensis* Fresen (Ranuncualaceae, Azo hareg)	Di	Cl	Hum	D	Wart	Tied fresh leaves on wart	W/H		
			Hum	D	Swelling, Wound	Fresh leaves squeezed and applied on swelling part		E008	X 426235 Y 1454125
			Hum	D	Ganglion cyst	Fresh leaves crushed and tied on affected part			
Unidentified (Sibta)	Di	H	Hum	O	Hepatitis	Fresh roots pounded and one is drunk	W	E009	X 417251 Y 1467237
*Brucea antidysenterica *J. F. Mill (Simaroubaceae, Waginos)	Di	T	Hum	O	Rectal prolapse	Fresh roots crushed mixed with “Difdif” (beer must) and then swallowed	W	E010	X 392703 Y 1462475
			Lk	D	Skin disorder of donkey	Soaking fresh/dried pounded roots within water in pot and wash the injured part			
			Lk	O	Dogs with rabies virus	Ground fresh/dried leaves finely, then mixed with milk and given to drink			
			Lk	O	Chicken’s feather loss	Fresh leaves pounded and smashed with hand and then applied on infected area like ointment			
*Clerodendrum myricoides* (Hochst.)(Lamiaceae, Misrich)	Di	H	Hum	O	Snake bite	Fresh/dried root is pounded, mixed with water and drunk	W	E011	X 379798 Y 1460659
			Hum	O	Coughing	Crushed fresh/dried roots, boiled with ginger and “abosida,” then filtered and drunk with milk			
*Crinum abyssinicum* (Amaryllidaceae, Yejib shinkuret)	Mo	H	Lk	O	To kill rat	Fresh bulbs pounded and gave it for rat to eat	W	E012	X 395554 Y 1462364
*Croton macrostachyus* Del. (Euphorbiaceae, Misana)	Di	T	Hum	O	Hepatitis	The fresh leaves macerated, mixed with honey and taken orally	W	E013	X 394252 Y 1459070
			Hum	O	Tapeworm	Fresh barks crushed and pounded; then eaten with boiled beans			
			Lk	O	Chicken disease	Fresh leaves pounded well and mixed with water and given to drink			
			Hum	D	Tinea corporis	Fresh leaves pounded well and applied on affected part			
			Hum	O	Snake bite	Chewing fresh barks and swallowing juice			
			Hum	D	Make baby fat	Young leaves crushed, mixed with water and with other herbs and showering body			
*Salvia nilotica* Vahl. (Lamiaceae, Hulegeb)	Di	H	Hum	O	Rabies	Dried root pounded, powdered and mixed with milk and taken orally	W	E014	X 379152 Y 1460758
*Unidentified* (–, yebere kolet)	Di	H	Hum	O	Sexual impotency	Dried roots powdered and mixed with honey; then taken orally			X 380233 Y 1461155
*Euclea racemosa* L. (Ebenaceae, Dedeho)	Di	Sh	Hum	O	Syphilis	Fresh/dried root is pounded and boiled with water then after cooling one goblets per day taken orally for 7 days	W	E015	X 402496 Y 1469130
*Euphorbia abyssinica* J. F. Gmel (Euphorbiaceae, Kulkual)	Di		Hum	D	Swelling	Applied fresh latex on swelling areas	W/H	E016	X 418304 Y 1455976
			Lk	O	Dog with rabis virus	Fresh latex mixed with milk and 1/3 of a “finjale” (cup) is given orally		E017	X 418304 Y 1455976
			Lk	D	Skin disorder of Donkey	Smeared fresh latex on horse body			
			Hum	D	Wart	Fresh latex rubbed on wart until it cures			
*Nicotiana tabacum* L. (Solanaceae, Timbaho)	Di	H	Lk	N	Leech	Crushed fresh leaves, mixed with water and given nasally	W	E018	X 377809 Y 1458448
			Lk	N	Cough	Pounded the fresh leaves, mixed with water and drench nasally			
*Ocimum lamiifolium* Hochst. ex. Benth. (Lamiaceae, Damekasie)	Di	H	Hum	O	Febrile illness	Fresh leaves crushed, poured into cup of coffee or alone, then taken orally	W/H	E019	X 380111 Y 1459682
*Olea europaea* sub spp.cuspidata L. (Oleaceae, woira)	Di	T	Hum	O	Abdominal pain	Dry Barks crushed, powdered and mixed with water and taken orally or fresh barks soaking in water and taken orally	W	E020	X 398407 Y 1466177
			Hum	O	Toothache	Chewing and holding the very young stem by teeth			
*Stephania abyssinica* (Dill. & A. Rich.) Walp (Menispermaceae, Yeayit areg/achebchabit)	Di	Cl	Hum	D	Febrile illness	Rubbed fresh leaves by hand and smeared on skin	W	E021	X 377800 Y 1458478
			Hum	O	Rabies	Fingertip size of fresh roots pounded combined with leaves of *Justicia* *schimperiana, Brucea antidysenterica and roots of Phytolacca* *dodecandra* then taken with whey			
			Hum	O	Swelling, wound	Pounded the fresh/dried root, mixed with water and about one cup is drunk			
*Ferula communis* L. (Apiaceae, Dog)	Di	H	Lk	O	Synchronizing cow	Crushed the fresh leaves, mixed with “Tella” and given orally	W	E022	X 394528 Y 1461815
			Hum	O	Hepatitis	Fresh roots dig out from three different places, pounded and mixed with local beer “Tella” then taken orally			
			Hum	O	Sexual impotency	Crushed fresh root and mixed with water; then” showered for 3 days			
			Hum	D	Headache	Ashes of shoots mixed with butter and applied on head			
			Hum	D	Circumcision	Dried stems heated with fire then pounded, mixed with butter and creamed circumcised penis			
			Hum	O	Rabies	Fresh roots rubbed with roots of *Rumex nepalensis*, then squeezed fluid into cup having latexes of Euphorbia ampliphylla, mixed with milk and then drunk half of a cup			
			Hum	O	Initiating cow ready to mate	Crushed fresh roots, mixed with salt and then fed to cow			
			Lk	O	Mich	Fresh roots crushed, pounded and mixed with water; then given orally			
*Brassica carinata *A. Br. (Brassicaceae, Gomen zer)	Di	H	Hum	O	Abortion	Fresh roots pounded and mixed with water; then drunk	H	E023	
*Clutia abyssinica *Jaub. and Spach. (Euphorbiaceae, Fiyelefej)	Di	H	Hum	O	Make someone to have illness	Fresh roots crushed, pounded then mixed with food and eaten	W	E024	X 383785 Y 1456730
			Hum	D	Wound	Dried roots powdered and applied on wound and wrap it with piece of cloth			
			Hum	O	Rabies	Powdered fresh/dried roots then taken orally			
			Hum	D	Dandruff	Fresh fruits pounded and applied on affected part			
			Lk	D	Cattle lice	Fresh leaves crushed, pounded and mixed with a little water then applied on skin			
			Hum	D	Tinea corporis	Fresh fruits pounded and applied on affected part			
*Malva verticillata* L. (Malvaceae, Yekebit lut/yesewlut)	Di	H	Hum	D	Bleeding, wound	Rubbed the fresh leaves and applied on topically	W	E025	X 429205 Y 1453958
			Lk	O	Weaken baby	Fresh stems pounded together with *Rumex nepalensis* and mixed with trigonella foenum, then given orally			
Unidentified (–, Dinbo)	Di	H	Hum	D	“Diro”	Crushed the fresh/dried roots together with leaves and smear on body	W	E026	X 432469 Y 1457375
			Hum	D	Circumcision	Rubbed the fresh leaves and tied on tips of circumcised penis			
			Lk	D	Atopic Dermatitis	Fresh leaves pounded and creamed on skin			
			Hum	D	Bleeding	Crushed the fresh leaves and then tied on affected part			
			Lk	O	Retained placenta	Soaking multiple fresh roots in water, adding a few salt and given orally			
			Hum	O	Cough	Roots crushed, pounded, mixed with water and taken orally			
*Ficus palmata* Forssk. (Moraceae, Beles/Qotilebele-s)	Di	T	Hum	D	Wound, bleeding	Fresh leaves pounded well and then applied on affected areas	W/H	E027	X 398434 Y 1465491
			Hum	D	Wart	The fresh latex is applied on wart			
*Ficus sur *Forssk. (Moraceae, Sholla)	Di	T	Hum	O	Ascariasis	Powdered the dried roots mixed with powdered roots of *Croton macrostachyus*and *Otostegia integrifolia* and mixed with honey and then taken orally	W	E028	X 392141 Y 1461833
*Carduus macracanthus* Sch.Bip. ex Kazmi (Asteraceae, Kosheshelie)	Di	H			To get more butter	Fresh roots tied together with roots of *Rumex nervosus* Vahl and *Solanum marginatum*, then inserted into a pot containing milk or tie on neck of pot	W	E029	X 417706 Y 1470814
			Lk	N	Mich	Fresh/dried roots pounded combined with *Rumex nepalensis*, *Verbasicumstelurum, Carduus schimperi*, Steganotaenia araliacea and *Hagenia abyssinica*; then mixed with water and drench nasally			
					To stop raining snow	Fresh roots chopped, combined with roots of *Solanum marginatum*,*Verbasicumstelurum* Murb, *Lobelia rhynchopetalum*, *Rumex nepalensis and Ferula communis* and then saying magical words on them for 7 days and tied on top of tree of village			
*Polygala abyssinica* Fresen (Polygalaceae, Yebabmedahnit)	Di	Sh	Hum	O	Snake bite	Fresh roots crushed and chewd	W	E030	X 398297 Y 1467214
*Phytolacca dodecandra* L 'Herit. (Phytolaccaceae, Endod)	Di	Cl	Hum	O	Abortion	Fresh root is crushed, pounded and mixed with water and taken orally	H	E031	X 377935 Y 1458357
			Hum	O	Body swelling	Fingertip size root crushed, pounded and drunk			
			Hum	O	Hepatitis	Fingertip size of fresh/dried root squashed mixed with water, then ¼ of cup drunk			
		Cl	Hum	O	Rabies	Crushed fresh/dried roots, pounded and mixed with water then drunk Or roots pounded together with roots of Euphorbia ampliphylla; then backed with Teff and eaten			
			Hum	O	Gastric enteritis	Pounded the fresh roots, mixed with water and then taken orally			
			Hum	O	Malaria	Squeezed the fresh leaves then 1/3 cup of juices taken orally			
*Rumex abyssinicus *Jacq. (Polygonaceae, Mekikeko)	Di	H	Hum	O	Abdominal pain	Pounded the dried fruits, boiled and taken as tea	W	E032	X 414922 Y 1456505
			Hum	O	Cough	Boiled fresh/dried roots then taken as tea			
			Hum	D	Skin fissure	Rubbed the fresh leaves and applied on leg			
			Lk	O	Cattle abdominal distension	Crushed fresh/dried root and mixed with water; then one litter is given to drink Or boil the crushed roots and stay away until it cools down; given orally			
			Hum	O	Hepatitis	Crushed the fresh/dried roots, boiled and then taken orally			
			Hum	O	Malaria	Ground the fresh leaves, mixed with broth of meat and taken orally			
			Lk	O	Retained placenta	Crushed the fresh roots and leaves, pounded mixed with soap and given orally			
*Rumex nervosus *Vahl. (Polygonaceae, Embacho)	Di	H	Hum	D	Psychiatric problem	Crushed the fresh roots, mixed with water then showering all bod parts	W	E033	X 413890 Y 1455273
			Hum	O	Abdominal pain	Young leaves crushed and hold it with teeth			
			Lk	N	Nasal discharge in sheep	Rubbed the fresh leaves and placed the fluids into nostril			
			Hum		To break black magic	Dried stems put on burning fire and fumigated the room when all family members are available			
			Hum	O	Intestinal parasite	Chewed the young leaves and then swallowing juices			
			Hum	D	Cleaning dead body	Cut fresh leaves and washed dead person within water			
			Hum	D/O	Hepatitis	Young leaves squeezed and applied on skin; then soldering it by warmed needle Or young leaves boiled and taken orally			
			Hum	D	Circumcision	Heated dried shoots with fire, pounded it then applied on penis			
					Keep house from wind	Crushed roots of *Kalanchoe petitiana* and Aloe steudneri, tied together, then rounding the house three times then put on ceiling			
			Lk	N	Cough	Crushed the fresh roots, mixed with goat milk and nasally			
			Hum	Op	Bleeding during birth	Pounded the dried roots with roots of *Verbasicumstelurum, Ferula communis, Stephania abyssinica* and *Otostegia integrifolia*; then soaked in water and spray to woman eye			
			Lk	N	MICH	Powdered the fresh roots put on fire and fumigate			
			Hum	D	Eczema	Roasted the leaves, powdered and mixed with butter then applied on the affected part			
			Lk	Op	Impaired eye	Squeezed the fresh young leaves, then applied on impaired eye			
			Hum	D	Burnt body	Heated the dried stem with fire, then powdered and applied on burnt area			
*Thalictrum rhynchocarpum *Dill. & A. Rich (Ranunculaceae, Sire bizu)	Di	H	Hum	O	Gastric enteritis	Crushed the fresh roots with *Bersama abyssinica*, *Verbasicumstelurum*, powdered it, then taken orally	W	E034	X 379807 Y 1460671
			Lk	O	Abdominal distension in cattle	Powdered the fresh/dried roots and mixed with water and given orally			
*Hagenia abyssinica* (Bruce) J.F.Gmel. (Rhamnaceae, Kosso)	Di	T	Hum	O	Tape worm	Pounded the fresh/dried fruits, mixed with water then drunk	W/H	E035	X 395821 Y 1462918
			Lk	O	Abdominal distension in cattle	Pounded the dried fruits, mixed with water and given orally			
			Lk	D	Broken leg	Tied the fresh leaves on the broken leg with aid leaves			
*Rosa abyssinica* Lindley (Rosaceae, Kega)	Di	Sh	Hum	O	Ascariasis	Ate fresh fruits before meal	W	E036	X 396272 Y 1463133
			Hum	O	Gastritis	Ate the fresh fruits			
*Osyris quadripartita *Decn. (Santalaceae, Keret)	Di	Sh			Evil spirits	Laid fresh leaves around fence	W	Eo37	X 396885 Y 1462679
					Make milk good taste	Fresh leaves heated on fire and fumigated milk containers			
*Helichrysum citrispinum* (Asteraceae, Yewalya eshoh)	Di	H	Lk	O	MICH	Crushed the fresh/dried roots and taken orally	W	E038	X 414431 Y 1464693
*Dodonea angustifolia* L.f. (Sapindaceae, Kitkita)	Di	Sh	Lk	O	Fattening cattle	Leafs dried, pounded and mixed with *Eleusine coracana* (“Dagussa”) within water and taken orally	W	E039	X 395558 Y 1462422
			Hum	D	Burnt body	Roasted the dried leaves, pounded then applied on burnt part			
			Hum	O	Gastric enteritis	Chewed three to four seeds by teeth and swallowed			
			Hum	O/N	COVID-19	Burn the dried stem with fire and fumigated			
			Hum	O	Malaria	Pounded the fresh fruits, mixed with honey, then swallowed			
			Hum	D	Wart	Fresh roots crushed, pounded and applied on wart; then soldering wart by heated stick of *Rumex nervosus*			
*Helichrysum horridum* (Asteraceae, Tifergina)	Di	H	Lk	Op	Impaired eye	Fresh roots pounded combined with leaves of *Inulaconfertiflora*, mixed with butter then dropped into cattle eye	W	E040	X 415013 Y 1465330
			Hum	D	Childs bedwetting	Peel the fresh bark and tied on child’s waist			
			Hum	O	Uterine myoma	Pounded dried roots with seeds of *Lobelia rhynchopetalum* and *Sesamum indicum*, then eaten			
			Hum	N/D	Psychiatric problem/tselay tsenay	Crushed the fresh roots, pounded with *Lobelia rhynchopetalum*, *Verbasicumstelurum*, *Ferula communis, Ruta chalepensis *and *Allium sativum*, then wrapped with cloth and smell or tied on neck			
*Withania somnifera *(L.) (Solanaceae, Gizewa)	Di	Sh	Hum	D	Malaria	Pounded fresh/dried roots and mixed with butter then applied topically	W	E041	
			Hum	O	Sexual Impotency	Pounded fresh/dried roots with roots of *Lobelia rhynchopetalum*, *Rumex nepalensis*, *Ferula communis, Clutia abyssinica, Thalictrum rhynchocarpum* and *Habenaria petitianaa* then Drunk with barley TELLA			
*Achyranthes aspera* Lam. (Amaranthaceae, Telenj)	Di	H	Hum	D	Tinea Unguniunm	Crushed fresh leaves then applied or rubbed on affected part	W	E042	X 393916 Y 1462249
			Hum	D	Bleeding during give birth	Powdered fresh/dried roots, wrapped by piece of cloth, then tied on waist by cotton thread			
		H	Hum	Ug	Bleeding during birth	Pounded fresh roots finely with roots of *Verbasicumstelurum* and then put it on female vagina			
			Lk	O	Impaired eye	Fresh leaves pounded, mixed with urine and then inserted to impaired eye			
			Hum	D	Wound, bleeding	Squeezed fresh leaves and dropped on bleeding and tied the pounded leaves on affected part			
			Hum	D	Burnt body	Fresh leaves crushed, pounded and applied on affected part			
			Hum	D	Tinea corporis	Fresh leaves are pounded and tied on affected area			
*Allium sativum *L. (Alliaceae, Nech shinkuret)	Mo	H	Hum	O	Coughing	Crushed the dried leaves, mixed with honey and One table spoon taken orally for 7 days	H	E043	X 392487 Y 1460525
			Hum	O	Toothache	Held the fresh/dried leaves by teeth			
			Hum	D	Evil eye	Fresh/dried bulbs pounded together with *Ruta chalepensis* and tied on neck			
*Aloe steudneri* (Schweinf Aloaceae, Ret)	Mo	H	Lk	O	Fattening cattle and sheep	The stem is cut into parts, mixed with salt and eaten	W	E044	X 420764 Y 1473448
			Lk	D	To get hair black	Fresh inner gel species applied on hair after washing			
			Hum	D	Bleeding, wound	Fresh inner gel of species removed and tied on affected part			
			Hum	O	Snake bite	Fresh latex of species squeezed and then eaten			
*Catha edulis Forsk* (Celastraceae, Chat)	Di	T	Hum	D	Evil spirit	Fresh leaves masticated and spitted out on the patient	W/H	E045	X 392857 Y 1459680
			Hum	D	Abdominal distension	Boiled fresh leaves, mixed with sugar and half of cup is drunk			
*Capsicum annuum *L. (Solanaceae, Qariya)	Di	H	Hum	D	Tinea versicolor	Fresh leaves pounded and applied on affected part	M	E046	X 379898 Y 1460801
			Hum	D	Wound	Crushed fresh leaved, pounded and applied on wound			
*Carduus schimperi* Sch. Bip. ex A. Rich. (Asteraceae, Yemdir eshoh)	Di	H	Lk	O	Bet with stick	The fresh root pounded, mixed with water and given orally	W	E047	X 393465 Y 1461989
			Hum	O	Abdominal pain	Dug out fresh roots from three distinct places, pounded, mixed with water, drunk			
			Lk	O/N	Mitat	Fresh roots crushed, mixed with water and given orally or through left nose Or heated the root and fumigating the smoke			
			Lk	O	Nasal discharge	Crushed the fresh/dried roots, pounded and mixed with water an given orally to sheep			
			Lk	O/N	Cough (Kuro)	The fresh/dried root is pounded and mixed with water then given orally and nasal			
			Lk	O	Getting cow to accept her calf	Fresh roots dig out from three places and crushed; then fed the cow			
			Hum	O	Dysentery	Fresh/dried roots powdered with roots of *Rumex nepalensis* then mixed with honey and taken orally			
*Cucumis ficifolius* A. Rich (Cucurbitaceae, Yemedir embuay)	Di	Cl	Hum	D	eczema	Powdered fresh roots, mixed with butter and creamed on the affected part until it cures	W	E048	X 395786 Y 1462429
			Hum	O	Hepatitis	Fresh roots pounded and mixed with water and taken orally			
			Hum	O	Abdominal pain	Powdered dried roots, mixed with water and then drunk			
			Hum	O	Abortion	Pounded fresh fruits and mixed with water, then drunk			
			Lk	O	Kofa	Fresh roots pounded and mixed with water; then drunk			
			Hum	D	Retained placenta	Cut fresh leaves by calling “let it down” seven times and tied on left waist			
*Datura stramonium* L. (Solanaceae, Astenagir)	Di	H	Hum	D	Dandruff	Fresh leaves rubbed and applied topically on head after washing	W/H	E049	X 376973 Y 1459043
			Hum	O	Toothache	Boiled the fresh fruits with barks of “SINICH” and then inhaled the steam orally or washed mouth with boiled solution			
			Lk	Op	Impaired eye	Rubbed fresh leaves and drop juices on eye			
			Hum	D	Make baby fat	Washed the body with young leaves			
			Hum	D	Tinea corporis	Squeezed fresh leaves and applied on affected area			
			Hum	D	Birth bleeding	Fresh roots dig out from seven distinct places and tied on waist with cotton thread			
			Hum	D	Wound	Fresh leaves pounded and applied on wound			
*Embelia schimperi* Vatke (Myrsinaceae, Enqoqo)	Di	T	Hum	O	Tape worm	Dried fruit is powered and dissolved by water; then half of cup is taken	W	E050	X 399385 Y 1465983
*Eucalyptus globulus *Labill. (Myrtaceae, Nech Bahirzaf)	Di	T	Hum	O	Common cold	Fresh leaves boiled and inhaled	H	E051	X 378946 Y 1457123
*Jasminum abyssinicum* L. (Oleaceae, Tenbelel)	Di	Cl	Hum	O	Abdominal pain	Rubbed the fresh bud by hands, mixed with few water and taken orally	W	E052	X 379185 Y 1461241
			Hum	O	Toothache	Fresh leaves crushed and hold by teeth			
			Hum	Op	Eye infection	Fresh leaves squeezed and applied for a few minutes			
			Hum	O	Urine retention	Fresh roots powdered along with powdered roots of *Zehneria* *scabra* and “SINCH,” mixed with water and drunk (1 cup)			
			Hum	O	coughing	Fresh roots pounded together with roots of *Verbasicumstelurum* and *Rumex nervosus *and mixed with water and half liter is drunk			
			Hum	O	Psychiatric disorder	Soaked fresh roots in water, with *Lobelia rhynchopetalum*, *Ferula communis,* Allophylus abyssinicus and Mahilda then washed bodies for 14 or 28 days depending on severity of disease			
*Kalanchoe petitiana* A. Rich (Crassulaceae, Endawula)	Di	H	Hum	O	Febrile illness	Boiled the pounded fresh root, mixed with sugar and drunk	W	E053	X 401389 Y 1468253
			Lk	D	Dislocated bone	Small opening is made in the dislocated area and then parts of fresh root are inserted to be opened for 12 h			
			Hum	D	Rectal prolapse	Fresh leaves warmed on fire along with *Kalanchoe petitiana* leaves then rubbed the back of patient			
			Hum	O	Tonsillitis	Fresh roots pounded, mixed with water and taken half of cup			
			Hum	D	Retained placenta	Rounded her abdomen by fresh roots and tied on waist with cotton thread			
			Hum	D	Burnt body	Fresh leaves warmed up by fire and rubbing the burnt area			
			Hum	D	Skin fissure	Fresh leaves warmed up by heat and rubbed the affected areas			
*Lepidium sativum* L. (Brassicaceae, Feto)	Di	H	Hum	O	Hypertension	Dried fruits pounded, mixed with water and taken orally	H	E054	X 394169 Y 1459533
			Hum	O	Malaria	Ate the pounded seed			
			Hum	O	Intestinal parasite	Dried seeds pounded and mixed with water then drunk one cup			
			Hum	D	Chalazion	Dried stems warmed up on fire and then soldering affected part			
			Hum	D	Wart	Warmed up the dried shoot with fire and put it on wart			
			Hum		Abortion	Ground the dried seed, mixed with water then taken orally			
*Lobelia rhynchopetalum* (Hochst) Hemsl (Lobeliaceae, Jibara)	Mo	H	Hum	O	Abdominalcramp	Fresh/dried roots pounded with water and taken 1/3 of a cup	W	E055	X 403662 Y 1467228
			Hum	N	Evil eye	Crushed fresh roots, mixed with *Artemisia abyssinica*then wrapped together in a piece of cloth and sniffed through nose Or roots crushed together with fruits of *Carissa spinarum*; then put on fire and fumigate			
			Hum	D/N	Febrile illness	Crushed and pounded the fresh/dried root and smeared on body or Smelling the crushed root			
			Hum	D	Wound	Dropped latex on wound			
			Hum	-	Epidemic disease	Dried leaves heated with fire and fumigated the door			
			Lk	N O	Cough (Kuro)	Heated the dried barks with fire with *Euphorbia ampliphylla*, then fumigated equines			
			Lk	O	Rabies (for Dog	Fresh/dried roots pounded together with fruits of *Phytolacca* *dodecandra* and roots of *Rumex nepalensis* then mix water; and one cup is drunk			
			Hum	D	eczema	Powdered dried fruit, mixed with honey and then applied topically			
			Lk	O	Nasal discharge in sheep	Applied fresh latex on affected part			
			Lk	O	Fattening sheep and ready to get pregnant	Crushed fresh leaves, mixed with salt and then fed to sheep			
*Allium cepa* L. (Amaryllidaceae, Key shinkurt)	Mo	H	Hum	O	Cough	Bulbs crushed, boiled and mixed with sugar; then drunk as tea	M	E056	X 394497 Y 1458316
*Otostegia integrifolia* Benth (Lamiaceae, Tinjit)	Di	H	Lk		Insect repellants	Fumigated the house by burning fresh stem	W	E057	X 393709 Y 1459876
			Hum	O	Syphilis	Pounded the dried root, mixed with water then taken orally			
					Keep flies away	Heated the fresh stems and leaves with fire and then fumigated the house			
			Hum	D	Rh factor	Crushed fresh/dried roots which dug out from seven places with *Clutia abyssinica* then tied on neck			
			Lk	N	Nasal discharge in sheep	Ground the fresh/dried leaves with leaves of *Olea europaea* subsp. *cuspidata*and roots of *Silenemacrosolen*, burn on fire, then fumigated the sheep and their shelter			
			Lk	N	Cough (Kuro)	Fresh/dried stems heated together with leaves on burning charcoal and fumigated			
			Hum	O	Abdominal pain	Fresh leaves pounded, mixed with water; then filtered out and half a size of cup is drunk			
			Lk	O/N	Epidemic/wetetie	Fresh/dried roots pounded and sprinkled on hot fire and fumigated			
*Rhamnus prinoides* L’Herit (Rhamnaceae, Giesho) *Rumex nepalensis* Spreng (Polygonaceae, Yewisha lut/lut)	Di	T	Lk	O	Abdominal distention in cattle	Pounded the dried leaves, mixed with malt and then given orally	W/H	E058	X 390781 Y 1460749
			Hum	O	Retained placenta	Ground the dried finely, mixed with *Linum usitatissimum*seed powder, mixed with water then drunk			
			Hum	O	Toothache	Held the fresh leaves by teeth			
			Lk	N	Leech	Crushed the fresh leaves, pounded and mixed with water then drench in nostril			
	Di	H	Hum	D	Bleeding, Wound	Fresh root is pounded, powdered and then applied on affected part	W/H	E059	X 441259 Y 1465528
			Hum	O/N	Kofa	Dug out fresh roots from three distinct places and pounded, then drench via nose and oral			
			Lk	O/N	Mad cow disease	Pounded fresh/dried leaves, mixed with water and then drench via left nose and ear			
			Lk	O	Abdominal distension in cattle	Crushed fresh roots and mixed with water then given orally			
			Hum	O	Febrile illness	Washed and pounded fresh roots, mixed with water, then taken orally			
			Hum	-	Retained placenta	Fresh/dried roots put into prepared hole at center of house, then boiled waters poured into hole			
			Hum	D	Ganglion cyst	Fresh roots crushed and tied on affected part until it cures			
			Lk	N	Mitat	Pounded the fresh root, mixed with water, given via left nose			
			Hum	D	Wart	Fresh roots pounded and rubbed the wart gently			
			Lk	N	“Yebetir”	Fresh roots crushed, mixed with water, then given orally			
			Hum &Lk	O	Urinary retention	Crushed and pounded the fresh root, then drunk with water			
			Hum	O	Toothache	Crushed the fresh leaves, then hold by teeth for few minutes			
			Lk	O	Retained placenta	Crushed and pounded fresh roots then given orally			
			Hum	O	Abdominal pain	Crushed and pounded the fresh roots, mixed with water, filtered, then drunk			
			Hum	O	Dysentery	Pounded the fresh roots with roots of *Achyranthes aspera* and rhizomes of ginger, then taken orally			
*Urtica simensis* Stedel (Urticaceae, Sama)	Di	H	Hum	O	Abdominal pain	Crushed fresh roots finely, then taken orally	W	E060	X 441183 Y 1465052
			Hum	D	Burnt body	Roots roasted, powdered and put on burnt body			
			Hum	D	Tinea versicolor	Cleaned fresh leaves, pounded and tied with piece of cloth on affected area			
			Hum	O	Appetite	Chopped and cooked fresh leaves, then ate with Injera			
		H	Hum	O	Intestinal infection	Cooked the fresh leaves and then ate			
			Hum	O	Yebetir	Fresh roots crushed, pounded and mixed with water and given orally			
			Hum	D	Evil eye	Boiled fresh roots then showered the body with the decoction			
			Lk	O	Emaciation in cattle	Fresh roots of species given to cattle to eat			
			Lk	O	Retained placenta	Pounded the fresh roots to make a solution, then certain amounts given orally			
*Verbasicum stelurum* Murb (Scrophulariaceae, Qutina)		H	Hum &Lk	O/N	Mich	Fresh root is pounded, mixed with water, filtered and given orally and via left nose	W	E061	X 397223 Y 1463508
			Hum	O	Abdominal pain	Pounded fresh roots and mixed with water; then drunk			
			Lk	N	KOFA	The fresh root is crushed and mixed with water; then drench to nostril			
					To get more yield/profit	The fresh roots with roots of *Rumex nervosus* and *Helichrysum horridum* put out side threshing floor			
			Hum	D	Bleeding during giving birth	Crushed the fresh roots and tied on waist			
			Hum	O	Burnt body	Roasted and crushed fresh roots and mixed with butter then creamed			
			Hum	N	Psychiatric problem	Dried roots are powdered and inserted to patient nose			
				-	For envy	Fresh roots crushed, mixed with water and sprinkling on burning fire, then fumigated house			
			Lk	O	Yebetir (betting with stick)	Fresh root is pounded together with roots of *Urtica simensis* and *Rumex nepalensis*, then mixed with water and given orally			
			Hum	D	Wound	Fresh/dried roots are roasted, powdered and mixed with butter, then applied over wound			
*Verbena officinalis* L. (Verbenaceae, Atuch)	Di	H	Hum	N	Migraine	Fingertip size of fresh/dried roots boiled and taken nasally	W	E062	X 393286 Y 1461719
			Hum	O	Tonsillitis	Dried roots crushed, pounded and mixed with water then drunk			
			Lk	O	Mich	Fresh roots pounded, mixed with water and then given orally			
*Zingiber officinale* Roscoe (Zingiberaceae, Zinjible)	Mo	H	Hum	O	Abdominal pain	Chewed fresh/dried rhizomes, then swallowed the juice	M		
			Hum	O	Constipation	Crushed the fresh/dried rhizomes, mix water; then taken orally			
			Hum	O	Toothache	Crushed dried rhizomes and hold by teeth for 5–10 min			
			Hum	O	Abdominal pain	fresh/dried rhizomes crushed and pounded with the roots of *Rumex nepalensis*, mixed with honey, then taken orally before meal			
*Euphorbia platyphyllos* L. (Euphorbiaceae, Tena demo/abay dem)	Di	H	Hum	D	Tinea versicolor (kukuasisha)	Squeezed the fresh latex and applied on affected part		E063	X 397043 Y 1463289
*Echinops kebericho *Mesfin (Asteraceae, Kebericho)	Di	H	Lk	N/O	Mich	sprinkling crushed roots on burning charcoal and fumigating	W	E064	X 431976 Y 1462322
			Hum	O	Evil eye	Pounded dried roots, mixed with water, then taken orally			
			Lk	N/O	Cough	Fumigated dried roots			
*Ruta chalepensis* L. (Rutaceae, Tenadam)	Di	H	Hum	D	Evil eye	Powdered dried roots with *Urtica simensis*, mixed with hyena`s liver then tie on neck Or roots powdered with bulbs of *Allium sativum* and leaves of *Rhamnus prinoides* and wrapped with piece of cloth, then smell and tied on neck	M	E065	X 393495 Y 1458371
		H	Hum	O	Abdominal pain	Pounded fresh leaves, mixed with water, filtered, then drunk			
			Hum	O	Toothache	Chewed the fresh fruit			
*Euphorbia tirucalli* L. (Euphorbiaceae, Qinchib)	Di	Sh	Hum	O	Wound	Fresh latex is applied on wound	W	E066	X 378956 Y 1460183
*Citrus aurantiifolia* (Christm.) Swingle (Rutaceae, Lomi)	Di	T	Hum	O	Hypertension	Fresh/dried fruits squeezed into cup, then taken orally	M	E067	X 397727 Y 1464922
			Hum	D	Athletes foot	The fresh fruit of *Citrus lemon* is squeezed and applied on affected areas for continuous days			
			Hum	D	Dandruff	Fluids from fruit smeared on head after washing			
*Rubia cordifolia* L. (Rubiaceae, Mencherur)	Di	Cl	Hum	O	Abdominal pain	Chewed fresh/dried roots, then swallowed its juice	W	E068	X 379867 Y 379867
			Hum	N	Eye infection	Powdered fresh/dried roots, mixed with butter; then melt with sun and filtered out with piece of clean cloth and half a cup is inserted into nose			
			Hum	O	Cough	Boiled the powdered fresh/dried root and drunk as tea			
			Hum	O	Tuberculosis	Dried roots powdered, mixed with honey and ate a cspoonful per day for seven days			
			Hum	O	Urine retention	Crushed dried roots, powdered, mixed with water, then taken orally			
			Hum	O/N	Headache	Powdered dried roots, mixed with butter and expose to sun for a few minutes and then place in nostril Or boiled roots, mixed with sugar, then taken as tea			
			Hum	N	Migraine	Boiled dried roots, then poured into nose			
*Vicia faba* L. (Fabaceae, Bakela)	Di	H	Hum	D	Boil	Crushed seven seeds by teeth and then put it on affected areas	H	Eo69	X 404208 Y 1463176
*Schefflera abyssinica* (Hochst. ex A.Rich.) Harms (Araliaceae, Getem)	Di	T	Hum	D	Eczema	Dried barks grinded, mixed with honey, then creamed over eczema	W	E070	X 395898 Y 1461946
			Hum	D	Syphilis	Dried barks pounded, mixed with honey and applied on penis			
*Grewia ferruginea Hochst. ex A. Rich* (Tiliaceae, Lenquata)	Di	Sh	Lk	O	Retained placenta	The fresh bark is pounded, mixed water then liquid extracts given orally within salt	W	E071	X 398678 Y 1466213
			Lk	O	Emaciation in cattle	Soaked fresh barks in water for one day, then given orally			
*Hordeum vulgare* L.(Poaceae,Gebs)	Di	H	Hum	O	Gastritis	Roasted the dried grain and then ate	H	E072	X 416906 Y 1470097
*Hypericum revolutum* (*Forssk.) Vahl (* Guttiferaceae, Amija)	Di	Sh	Hum	O	Febrile illness	Fresh leaves boiled together with leaves of *Thymusschimperi* and *Zehneria* *scabra*, then inhaled the steam	W	E073	X 396459 Y 1462980
*Juniperus procera* Endl. (Cupressaceae, Yehabesha tid)	Di	T	Lk	O	Protecting livestock from Hyena preying	Fresh/dried roots pounded with roots of *Kalanchoe petitiana*, *Osyris quadripartita*, *Stephania abyssinica* and *Clutia abyssinica,* mixed with dungs of Hyena and black barely; then fed to cattle	W/H	E074	X 394954 Y 1462141
*Justicia* *schimperiana* (*Hochst.ex* *Nees) T. Anders* (Acanthaceae, Simeza/sensel)	Di	Sh	Hum	O	Rabies	Fresh leaves pounded with leaves of *Momordica foetida* and *phytolacca* *dodecandra,* mixed with water, then taken orally	W	E075	X 394335 Y 394335
			Lk	N	Leech	Fresh leaves pounded and mixed water, then drench in nostril			
			Lk	O	Circling disease in sheep	Fresh leaves pounded with leaves of *Inula confertiflora,* mixed with water, then given orally and sprayed in the house			
*Zehneria* *scabra* (Linn. f.) Sond. (Cucurbitaceae, Aaregresa)	Di	Cl	Hum	N/O	Febrile illness	Boiled the fresh leaves and stem, then inhaling the steam through mouth and nose	W/H	E076	X 392147 Y 1460762
			Hum	O	Abdominal pain	Fresh roots crushed and pounded with rhizomes of ginger and 1/3 of cup taken orally			
			Hum	D	Arthritis	Boiled fresh leaves and stem, then showered the body with the decoction			
			Hum	D	Tinea Unguniunm	Crushed and pounded the fresh leaves, then tied on the affected parts			
			Hum	O	Febrile illness	Fresh leaves boiled in water, mixed with suagr and taken orally 1/3 of a cup			
			Hum	Op	Eye cellulitis	Boiled the young fresh leaves with water having sand, then fumigated the steam with eye			
*Momordica foetida* Schumach. (Cucurbitaceae, *Yekura Hareg*)	Di	Cl	Hum	D	Eczema	Pounded fresh leaves and applied on affected area	W	E077	X 414219 Y 1461839
*Silene macrosolen* Steud. ex A. Rich (Caryophyllaceae, Wogert)	Di	H		-	To make clothes good smell	Crushed dried roots withleaves of*Otostegia integrifolia* and *Securidaca longepedunculata*, sprinkled on burning charcoal then fumigated	W	E078	X 394682 Y 1461102
			Hum	O	Febrile illness	Fresh/dried roots pounded, boiled and the steam is inhaled			
		H	Lk	O/N	Lymphadenitis in sheep	Dried roots boiled combined with roots of *Echinops kebericho,* then fumigated to sheep			
			Hum	O	Abdominal pain	Dried roots crushed, pounded and mixed with water then drunk			
*Cyphostemma cyphopetalum* (Fresen.)Wild & Drummond (Vitaceae, Yejib hareg)	Di	Cl	Hum	D	Wound	Dried roots powdered, mixed with butter, then applied over wound	W	E079	X 395836 Y 1463131
*Dombeya torrida* (J.F. Gmel) Bamps (Malvaceae, Wulkfa)	Di	T	Lk	D	Broken bone	Tied the broken part by fresh peel of bark	W	E080	X 379595 Y 1458643
*Inula confertiflora* A. Rich. (Asteraceae, Woinagefet)	Di	Sh	Lk	Op	Impaired eye	Fresh leaves are rubbed by hand and droplets are added into eye	W	E081	X 426163 Y 1454153
			Lk	D	MICH	Dried shoots warmed on fire then used while solderingcattle			
			Lk	D	Cogh (‘Kuro”)	Fresh leaves pounded together with leaves of *Helichrysum citrispinum* and mixed with water, then given nasally			
		Sh	Hum	D	Burnt body	Fresh leaves crushed, squeezed, then applied on affected part			
*Satureja Simensis* (Benth.) Briq. (Lamiaceae, Lomishet)	Di	H	Hum	D	Baby weight loss	Crushed the fresh leaves and used as body wash	W	E082	X 426094 Y 1454125
*Thymus schimperi* Ronniger (Lamiaceae, Tosign)	Di	H		-	Make milk sweet taste	Heated the fresh/dried stems and leaves on fire, then fumigating milk containers	W	E083	X 394676 Y 1462586
			Hum	O	Arthritis	Pounded fresh, soaked in water, then employed bath			
*Urera hypselodendron* (Hochst.) ex A. Rich. (Urticaceae, Lankiso)	Di	Cl	Lk	O	Retained placenta	Juices extract from crushed fresh stem, mixed with water, then given orally	W	E084	X 399648 Y 1464531
*Stereospermum kunthianum* Cham. (Bignoniaceae, Zana)	Di	T	Hum	D	Bleeding after delivery	The fresh leaves are crushed and tied on females leg	W	E085	X 380435 Y 1460365
*Chenopodium ambrosioides*L. (Chenopodiaceae, Amedmado)	Di	H	Hum	D	Tinea corporis	Fresh leaves pounded and tied on affected part by plastic for 3 days	W	E086	X 381328 Y 1460717
			Hum	D	Eczema	Dried leaves are roasted and powdered, then mixed with honey, then applied on affected part until it cures			
*Plantago lanceolata* L. (Plantaginaceae, Wenberet)	Di	H	Lk	N	Cough (Kuro)	The whole fresh plant is heated on burring fire, then fumigated Or fresh leaves crushed, mixed with water and drench into nostril	W	E087	X 393421 Y 1462146
			Hum	D	Wound, bleeding	Squeezed the fresh leaves and tied by piece of cloth			
*Hibiscus micranthus* Lif (Malvaceae, Yebekilo changer)	Di	H	Lk	O	Yebetir	The fresh root is pounded and mixed with water, then given through left ear	W	E088	X 379069 Y 1461011
			Hum	D	Lifie	Fresh leaves are crushed, mixed with butter and tied on affected areas			
			Hum	D	Wound on neck	The fresh leaves are crushed, mixed with butter, then applied on the swelling as bandage			
*Impatiens tinctoria* A. Rich. (Balsaminaceae, Gusheret)	Di	H	Hum	D	Arthritis	Tubers of the plant pounded, boiled and reserve for 2 days; then put hands into solution for few minutes	W	E089	X 400404 Y 1468276
			Lk	O	Emaciation in cattle	Tubers of plant crushed, pounded and fed to animal			
*Kosteletzka begonifolia* (Ulbr.) Ulbr. (Malvaceae, Yemegerem)	Di	H	Hum	O	Body swelling	Fresh root is pounded, mixed with water and taken orally	W	E090	X 399180 Y 1468584
			Lk	O	Body swelling on dewlap	Fresh root is pounded, mixed with “TELLA” (local beer), then given orally			
			Hum	O	Scorpion bite	Chewed the fresh roots and held by teeth			
			Hum	O	Abortion	Crushed and pounded fresh roots, then take orally with water			
*Maesa lanceolata* Forssk (Myrsinaceae, Siwarya/Shiwarya)	Di	T	Lk	O	Leech	Crushed the fresh leaves, mixed with water, then given orally or added into rivers which harbors Leech	W	E091	X 377865 Y 1458555
*Coffea arabica* L. (Rubiaceae, Buna)	Di	Sh	Hum	D	Injuries shot by gun	Dry seeds are roasted, powdered and then applied on injured area	M	E092	
*Solanum marginatum* L.F. (Solanaceae, Embuay/Yedega embuay)	Di	Hb	Hum	Ug	Unwilling of cow to milking	Fresh fruit is crushed, mixed with butter and inserting through vagina	W	E093	X 390061 Y 1460551
			Hum	D	Injuries by gun shot	Dry leaves are pounded, powdered, then applied on injured part			
			Hum	D	Tinea Unguniunm	Fresh fruits pierced and entering affected fingers into hole of the fruit			
			Hum	D	Tonsillitis	The fresh root is crushed and drunk with water			
			Lk	D	Sheep bite by wolf	Juice of fresh fruits dropped over injured part			
			Lk	N	Sheep nasal discharge	Fluids from fruit mixed with milk and given nasally			
			Hum	O	Toothache	Crushed the fresh root and held by teeth for 5–10 min			
			Hum	D	Tinea versicolor	Collected juice from fresh fruits mixed with butter, applied on affected part			
*Securidaca longepedunculata* Fresen. Polygalaceae, Temenahay/Etse-menahe)	Di	T	Hum	-	Good cloth smell	Fresh/dried roots pounded and powdered, then used to wash clothes	W	E094	X 379481 Y 1461090
			Lk	N/O	MICH	Crushed dried roots, sprinkled on burning fire, then inhaled the smoke			
			Hum	O	Evil eye/“Yeje seb”	Crushed the fresh/dried roots, sprinkled on burning charcoal and then inhaled the smoke			
*Eleusine coracana* (L.) Gaertn. (Poaceae, Dagusa)	Di	H	Hum	O	Dysentery	Ate as porridge	H	E095	X 378777 Y 1460836
*Echinops macrochaetus* Fresen (Asteraceae, Dindero)	Di	H	Hum	O	Evil eye/“Yejeseb”	Crushed the fresh roots with *Solanum marginatum*, *Asparagus africanus*,*Allium sativum*, *Lepidium sativum* and *Crinum abyssinicum,* mixed with water, then taken orally	W	E096	X 426821 Y 1454867
*Haplocarpha schimperi* (Sch.Bip.) Beauv (Asteraceae, Wozber)	Di	H	Hum	D	Tinea versicolor	Squeezed the fresh leaves, then applied on affected part	W	E097	X 1454867 Y 1468027
*Euphorbia ampliphylla* Pax (Euphorbiaceae, Kulkual)	Di	T	Hum	D	Cancer	Fresh latex is applied on the affected body part	W	E098	X 418684 Y 1456590
			Lk	D	Scabby disease in sheep	Fresh latex is applied on mouth			
			Hum	D	Wart	Fresh latex applied on wart topically			
			Hum	O	Intestinal parasite	Smeared the latex on traditional cake pan, thenate			
			Lk	O/N	Cough	Heated the dried bark with fire, then fumigating the animal			
*Prunus persica* (L.) Batsch (Rosaceae, Kok)	Di	T	Lk	N	Leech	Crushed the fresh leaves, soaked in water and filtered out, then the solution is given through nose	H	E099	X 379577 Y 1457612
			Lk	O	Abdominal distension in cattle	Fresh leaves pounded, mixed with water and then given orally			
*Girardinia bullosa* (Steud.) Wedd. (Urticaceae, Duvara/Avervara)	Di	H	Hum	D	Burnt body	Crushed and squeezed the fresh root, then tied with piece of cloth by appling on burnt area	W	E100	X 377459 Y 1458640
*Clausena anisata* (Willd.) B (Rutaceae, Limuch)	Di	Sh	Hum	O	Hepatitis	Crushed the fresh leaves, mixed with honey, then ate for 3 or 7 days	W	E101	X 391569 Y 1461877
*Solanum aethiopicum* L. (Solanaceae, Komodoro)	Di	H	Lk	O	Leech	Crushed and pounded the fresh leaves, mixed with water, then drench in nostril	W	E102	X 379707 Y 1461580
*Vernonia rueppellii* Sch.Bip. ex Walp. (Asteraceae, Shutenie)	Di	T	Hum	D	Bleeding	Crushed the fresh leaves and tied on injured body	W	E103	X 396078 Y 1463048
			Hum	D	Bleeding	Roasted and powdered the fresh leaves, then applied on affected part			
*Lycopersicon esculentum* Mill. (Solanaceae, Timamtim)	Di	H	Lk	O	Leech	Crushed the fresh leaves, mixed with water and filtered, then given orally	W	E104	X 411060 Y 1464571
*Erythrina abyssinica* Lam. ex DC (Fabaceae, Aveterie)	Di	T	Hum	D	Infected wound	Dried roots are powdered and applied over wound	W	E105	X 395903 Y 1461963
*Myrica salicifolia* A. Rich (Myricaceae, Sinch)	Di	T	Hum	O	Abdominal pain	Powdered the fresh bark, mixed with honey, then swallowed	W	E106	X 400387 Y 1464014
			Hum	N	Evil eye	Dried barks are powdered with bulbs of *Allium sativum* and then smell			
			Hum	O	Toothache	Removed the outer pat of fresh bark, then holding bark by teeth			
*Ricinus communis* L. (Euphorbiaceae, Bulka)	Di	Sh	Hum	D	Wound	Powdered the dried roots and then applied over wound	W	E107	X 392913 Y 1460840
*Cucurbita pepo* L. (Cucurbitaceae, Duba)	Di	Cl	Hum	D	Headache	The fresh fruit is pounded and cooked, applied on head after cooling, then wrapped with kerchief	H	E108	X 397942 Y 1467057
*Ficus vasta* Forssk (Moraceae, Warka)	Di	T	Hum	O	Infertility	Fresh/dried barks crushed and soaked with water along barks of *Carissa spinarum*, *Croton macrostachyus* and roots of*Securidaca longepedunculata* and then wash the woman body by solution for 7 days	W	E109	X 398320 Y 1465707
*Olea europaea* subsp. cuspidata (Wall. ex G.Don) Cif. (Oleaceae, Woira)	Di	T	Hum	N	Nasal discharge in sheep	Ground the fresh stem with roots of *Silene macrosolen*, burn on fire and then fumigated their shelter and sniffed nasally		E110	X 397807 Y 1463225
*Cyperus dichroostathyus* A.Rich. (Cyperaceae, Giramta)	Mo	H	Lk	Dm	Retained placenta	Immersed the fresh stem into water and then tied on tip part of placenta in one side; and the mortar on other side, then pull down with mortar (wood made)	W	E111	X 395269 Y 1462639
*Solanum* *incanum* L. (Solanaceae, Yeqola embuay)	Di	H	Hum	N	Leech	Juice extract from fresh fruit is given nasally (one cup)	W	E112	X 397199 Y 1465679
				-	To get more yield/profit	Crushed the fresh root and placed around threshing floor			
			Hum	D	Wound	Squeezed the fresh fruit and applied the juice on wound			
			Lk	D	For impaired eye	Pounded the fresh leaves by adding water, then inserting into cattle’s eye			
*Kniphofia foliosa* Hochst (Asphodelaceae, Ayineba)	Mo	Hb	Hum	D	Tonsillitis (infant)	Pounded the fresh leaves with malt, mixed with mother’s saliva, then placed on baby’s head	W	E113	X 418180 Y 1472525
			Hum	N	Cough	Crushed the fresh leaves with leaves of *Rumex nervosus*, mixed with water, then drench in nostril			

Key: List of medicinal plants used for treating ailments in the study area, with scientific name, family, local name, Classification (Di = dicot, Mo = monocots) habit (Ha), shrub (Sh), tree (T), herb (H), climber (Cl), seed (Se), fruit (Fr), shoot (Sh), source, condition of preparation (fresh/F and dry/D), rout of administration (Ra), oral (O), dermal (D), nasal (N), optical (Op), urogenital (Ug).

## Abbreviations

CNS: Central nervous system
DMR: Direct matrix ranking
EMA: Ethiopian Meteorological Agency
FGD: Focus group discussion
FL: Fidelity level
ICD: International classification of diseases
ICF: Informant consensus factor
ISE: International society of ethnobiology
IUCN: International Union for Conservation of Nature
SMNP: Simien Mountains National Park
UNDP: United Nations Development Programme
UNESCO: United Nations Educational, Scientific and Cultural Organization
